# Comparative Proteomics of Oxalate Downregulated Tomatoes Points toward Cross Talk of Signal Components and Metabolic Consequences during Post-harvest Storage

**DOI:** 10.3389/fpls.2016.01147

**Published:** 2016-08-09

**Authors:** Kanika Narula, Sudip Ghosh, Pooja R. Aggarwal, Arunima Sinha, Niranjan Chakraborty, Subhra Chakraborty

**Affiliations:** National Institute of Plant Genome ResearchNew Delhi, India

**Keywords:** post-harvest storage, 2-DE coupled mass spectrometry, comparative proteomics, protein network, tomato fruit, shelf-life

## Abstract

Fruits of angiosperms evolved intricate regulatory machinery for sensorial attributes and storage quality after harvesting. Organic acid composition of storage organs forms the molecular and biochemical basis of organoleptic and nutritional qualities with metabolic specialization. Of these, oxalic acid (OA), determines the post-harvest quality in fruits. Tomato (*Solanum lycopersicum*) fruit has distinctive feature to undergo a shift from heterotrophic metabolism to carbon assimilation partitioning during storage. We have earlier shown that decarboxylative degradation of OA by *Fv*OXDC leads to acid homeostasis besides increased fungal tolerance in E8.2-OXDC tomato. Here, we elucidate the metabolic consequences of oxalate down-regulation and molecular mechanisms that determine organoleptic features, signaling and hormonal regulation in E8.2-OXDC fruit during post-harvest storage. A comparative proteomics approach has been applied between wild-type and E8.2-OXDC tomato in temporal manner. The MS/MS analyses led to the identification of 32 and 39 differentially abundant proteins associated with primary and secondary metabolism, assimilation, biogenesis, and development in wild-type and E8.2-OXDC tomatoes, respectively. Next, we interrogated the proteome data using correlation network analysis that identified significant functional hubs pointing toward storage related coinciding processes through a common mechanism of function and modulation. Furthermore, physiochemical analyses exhibited reduced oxalic acid content with concomitant increase in citric acid, lycopene and marginal decrease in malic acid in E8.2-OXDC fruit. Nevertheless, E8.2-OXDC fruit maintained an optimal pH and a steady state acid pool. These might contribute to reorganization of pectin constituent, reduced membrane leakage and improved fruit firmness in E8.2-OXDC fruit with that of wild-type tomato during storage. Collectively, our study provides insights into kinetically controlled protein network, identified regulatory module for pathway formulation and provide basis toward understanding the context of storage quality maintenance as a consequence of oxalate downregulation in the sink organ.

## Introduction

Post-harvest storage has profound impact on nutritional quality and economic loss due to patho-stress in food crops that is governed by combinatorial properties of sensorial attributes, organoleptic parameters and physiological characteristics. Indeed, these phenomenon are coordinated by a complex network of endogenous and exogenous cues. Physiological and biochemical reprogramming during post-harvest storage in the sink organ favors formation of multifaceted and genetically programmed features that culminates in changes in color, texture, flavor, and aroma (Carrari and Fernie, 2006; Wang et al., 2009a; Pegoraro et al., 2015). These irreversible regulatory processes are often associated with abscission and softening, timing of which is determined by organ sensitivity to hormones and nutritional composition (Davuluri et al., 2005). Besides, series of developmental transitions that serve as a fingerprint for sensory and quality trait are controlled by key genetic and regulatory storage related factors (Marondedze et al., 2014). Genetic switch associated with post-harvest associated fruit softening is instrumental to maintain the quality attributes and shelf life. Generally, reduction in fruit firmness due to softening is accompanied by frequent pathogen attack. Furthermore, texture and mechanistic behavior also contribute to changes in cellular turgor, permeability to pathogen attack, morphology related to fruit softening and decaying during storage (Brummell and Harpster, 2001). Despite the fact that there are many studies concerning genetic control and spatial regulation of fruit firmness, our current knowledge about the molecular determinants at translational level remains unknown.

Enhancing the shelf life of food crops, including fruits thus represents an urgent worldwide goal. In the developed countries, approximately 10–30% of harvested fruits is lost during post-harvest storage (Food and Agriculture Organization of the United Nations; http://faostat.fao.org), while in developing countries this loss exceeds over 30–50% annually (Salunkhe et al., 1991; Zhulong, 2013). Tomato (*Solanum lycopersicum*) ranks first among fruits and vegetables, with 16% of the total production worldwide. It is also the most studied fleshy fruit with ease of cultivation, short generation time, a small sized diploid genome, and good tolerance to interspecific crosses (Klee and Giovannoni, 2011). Although, approximately 5 million ha are utilized in production yielding 19.2 kg per habitant, loss due to post-harvest storage is still 10% (Food and Agriculture Organization of the United Nations; http://faostat.fao.org).

Metabolic state and feedback regulation of low molecular weight organic acids influence the sensorial attributes and organoleptic properties of fruit. As primary metabolic by-products, organic acids are metabolically active solutes in cellular osmoregulation and acid balance. Moreover, quantity and quality of organic acids is known to influence fruit traits (Causse et al., 2002). Organic acids comprise about 15% of the dry content of fresh tomatoes. Organic acids accumulation in tomato is determined by the feedback regulation of biosynthesis, degradation and storage. Several carboxylic acids, including citric, malic, tartaric, succinic and oxalic acid are known to impact the quality of tomato fruit (Petro-Turza, 1987). Research directed towards the role of oxalic acid (OA) in post-harvest storage has allowed advances in our understanding of acid homeostasis and metabolic regulation in climacteric and non-climacteric fruits (Zheng and Tian, 2006; Zheng et al., 2007a,b; Wang et al., 2009b; Wu et al., 2011). Furthermore, OA is known to degrade or weaken the cell wall due to chelation of Ca^2+^ (Bateman and Beer, 1965). Our recent studies have demonstrated that downregulation of OA by decarboxylative degradation enhance the organoleptic parameters in addition to increased resistance against necrotrophic fungal pathogens in tomato (Chakraborty et al., 2013; Ghosh et al., 2016). Despite exhaustive information regarding pathways involved in the tomato fruit maturation and shelf life maintenance, few detailed studies have been conducted to analyse dynamic relation of fruit quality trait assessment and organic acid fluxes at translational level during post-harvest storage.

Here, we aim to investigate the underlying mechanism of low oxalate regulated post-harvest storage quality in fruit. This study provides a detailed framework of protein patterning in wild-type and E8.2-OXDC tomato fruit in temporal manner till 120 h during post-harvest storage. Attention was focused to identify the most variable proteins related to reduce oxalic acid level and associated with increased texture and firmness. Further, we combined the quantitative models describing protein expression changes and correlation network in response to oxalic acid downregulation to build a set of protein subnetworks affected during post-harvest storage. Our data highlights for the first time the mechanistic correlation between oxalic acid downregulation and maintenance of textural properties in storage organ, fruit in particular.

## Materials and methods

### Plant material, storage and experimental design

To overexpress *Fv*OXDC in tomato fruits, the coding sequence of OXDC were cloned under the regulation of the ethylene-inducible fruit-specific promoter E8, producing the plasmid pS8.2 (Chakraborty et al., 2013). Transgenic tomato genotype (E8.2-OXDC) transformed with the construct pS8.2, downregulating oxalic acid alongwith the wild-type genotype tomato variety Pusa Ruby were used in this study. Wild-type and E8.2-OXDC tomato plants were grown in an experimental field of National Institute of Plant Genome Research in the month of October during the growing season. In brief, seeds were sown in a mixture of soil:vermiculite [3:1 (v/v)] and seedlings were transplanted to commercial tomato cultivated soil at 3–4 leaves stage. Equivalent-sized red ripe fruits were harvested from three randomized plots. Full sized fruit 4–5 days past color break are red referred as red ripe stage. Each biological replicate consisted of nine fruits of the red ripe stage from three different plants. The freshly harvested fruits were stored at room temperature under high humidity to allow post-harvest storage response till 120 h. Fruits were collected at different time points (0, 24, 48, 72, 96, and 120 h) during post-harvest storage. Tissues were immediately frozen in liquid nitrogen, ground to a fine powder, and stored at −80°C until further use.

### Textural analysis

Fruit firmness was determined using TA-XT Plus (Stable Microsystems). Each fruit was analyzed with a 75 mm-wide P75 compression plate. A speed of 2 mm/s was used to compress fruit by 4 mm with a compression probe of 4.5 cm in diameter. The firmness was defined as the response force to a 5 × g applied force. Eight fruits were used for each measurement. The significance was calculated by unpaired *t*-test.

### Measurement of membrane leakage

Eight discs (3–4 mm thickness and 15 mm diameter) from 4 fruits at the equatorial region (two discs per fruit on opposite region) were rinsed and incubated in 50 ml of distilled water for 4 h, and then an initial electrolyte leakage was monitored with a conductivity meter (1601E, Electronics India, India). Each sample was continued rinsing for 2 h after being boiled for 5 min, and then a final electrolyte leakage (total electrolyte) was monitored again. Relative leakage rate was defined as percent of initial electrolyte. The experiments were carried out in triplicates.

### pH measurement

Ten gram of fresh tissues from 4 equivalent sized red ripe fruit was homogenized with 25 ml distilled water and filtered, and then pH of the solutions was measured with a pH meter (Pinnacle 530 pH meter, Cole-Parmer, USA) at 25°C. The experiments were carried out in triplicates.

### Lycopene assay

Total lycopene was measured by the method of Perkins-Veazie et al. (2001). In brief, approximately 2 g of wild-type and E8.2-OXDC fruits were weighed and mixed with 5 ml of 0.05% (w/v) butylated hydroxytoluene (BHT) in acetone, 5 ml of 95% ethanol, and 10 ml of hexane. Purees were stirred on a magnetic stirrer during sampling and centrifuged at 10,000 g for 5 min at 4°C and supernatant was discarded. After shaking, 3 ml of deionized water were added to each vial and the samples were shaken for an additional 5 min on ice. The vials were then left at room temperature for 5 min to allow for phase separation. The absorbance of the upper hexane layer was measured at 503 nm blanked with hexane. The assay was performed in triplicates. The lycopene content of each sample was then estimated at 503 nm.

### Pectinesterase assay

Assay was performed according to Rouse and Atkins (1955). In brief, 500 mg of wild-type and E8.2-OXDC fruit tissues were ground in liquid nitrogen and homogenized in 20 ml of 0.2 N NaCl in a mortar. The homogenate was transferred into 50 ml of 1% citrus pectin (Sisco Research Laboratories Pvt. Ltd., India) in 0.2 N NaCl. The pH was adjusted to 7.6 by 0.2 N NaCl and maintained at the same level for 10 min under pH meter by dropping 0.01 N NaOH at room temperature. The assay was performed in triplicates. Pectinesterase activity was expressed as μmoles g^−1^ fresh wt.

### Estimation of organic acids

Organic acid content was determined from wild-type and E8.2-OXDC tomato till 120 h with respective kit using colorimetric assay according to manufacturer's protocol. (Biovision, USA). In brief, oxalic acid, citric acid, and malic acid were estimated with 15 mg of fruit ground in liquid nitrogen and homogenized in 600 μL of ice-cold assay buffer provided in the respective kit. The homogenate was incubated for 10 min on ice and centrifuged at 10,000 g for 5 min. The assays were performed using 100 μL of fruit extract in triplicates. The calibration curve was constructed using malate, citrate and oxalate as standards. Concentration of oxalic acid and citric acid was determined for absorbance reading at 450 nm. For malic acid absorbance was taken at 570 nm.

### Fruit protein extraction, 2 DE and image analysis

Soluble proteins were isolated from stored whole tomato fruits according to previously published method (Chakraborty et al., 2013). In brief, 2.5 g of frozen whole fruit tissue powder was homogenized in 3 volumes of extraction buffer containing 700 mM sucrose, 500 mM Tris-HCL pH 7.5, 100 mM KCL, 50 mM EDTA, 2% [v/v] β-mercaptoethanol and 1 mM PMSF by vortexing 15 min on ice. The total proteins were recovered by phenol extraction method. The mixture was vortexed for 10 min and centrifuged at 10,000 g at 4°C and the soluble proteins were recovered as supernatant. Proteins were then precipitated by addition of five volumes of 100 mM ammonium acetate in methanol overnight at −20°C. Precipitated proteins were centrifuged at 10,000 g for 30 min and the protein pellets were washed once with ice-cold methanol and three times with ice-cold acetone, air dried and resuspended in 2-D rehydration buffer [9 M urea, 2 M thiourea, 4% (w/v) CHAPS, 20 mM DTT, 0.5% (v/v) Pharmalyte (pH 4–7) and 0.05% (w/v) bromophenol blue]. The supernatant containing the soluble proteins was used for 2-DE. Protein concentration was determined by the 2-D Quant kit (GE Healthcare) using bovine serum albumin (BSA) as standard. Protein samples (300 μg) were loaded onto IPG strips (Immobiline DryStrip pH 4–7 NL, 13 cm; GE Healthcare Biosciences) by in-gel rehydration, and isoelectric focusing was carried out using IPGphor system (Amersham Biosciences, Bucks, U.K.) at 20°C for 35,000 Vh with current limit set to 50 μA/strip. The protocol for IEF was as follows: 150 V for 3 h, 400 V for 2 h, 3500 V for 2 h and a final gradient step of 8000 V for 2 h, for a total accumulated voltage of 35,000 Vh. The focused strips were subjected to reduction with 1% (w/v) DTT in 10 mL of equilibration buffer [6 M urea, 50 mM Tris-HCl (pH 8.8), 30% (v/v) glycerol and 2% (w/v) SDS], followed by alkylation with 2.5% (w/v) iodoacetamide in the same buffer. The strips were then loaded on top of 12.5% SDS-PAGE for second dimension separation (Chakraborty et al., 2013). Gels were stained with Silver Stain Plus kit (Bio-Rad) and scanned with a Bio-Rad Fluor-S system. Gel images were analyzed with PDQuest 7.2.0 (Bio-Rad). For each time point three 2-DE gels representing three biological replicates were used for the data analysis. The correlation coefficient has been maintained to at least 0.8 between the replicate gels. The detailed data analyses were carried out as described previously (Chakraborty et al., 2013). To compare spots across gels, a matchset representing a “standard image” of three replicates was created from six time points. The high quality spots (quality score >30) were used to calculate the median value for a given spot, and the obtained value was used as the spot quantity on the standard gel. Next, for comparison, protein spots observed in each time point were normalized to “total density in gel image” mode and spots were manually annotated. The spot volumes were further normalized using three unaltered protein spots across all the gels. The significantly altered (Log_2_ > 2.5, *p* < 0.05) spots with more than 2.5-fold change in abundance were selected for identification (Supplementary Tables S1–S3). All statistical analyses were performed as explained in the “Statistical Analysis” section.

### Protein digestion and MS analysis

The protein spots were mechanically excised from the gels, destained and trypsin digested prior to MS analysis according to standard technique (Casey et al., 2005). Trypsinolyzed protein spots were loaded onto a C_18_PepMap100 column (3 μm, 100 Å, 75 micron ID_15 cm) at 300 nL/min (LC Packings) and separated with a linear gradient of water/acetonitrile/0.1% formic acid (v/v) and analyzed by electrospray ionization using an ultimate 3000 nano HPLC system (Dionex) coupled to a 4000 Q-TRAP mass spectrometer (Applied Biosystems). The peptides were eluted with a gradient of 10–40% acetonitrile (0.1% formic acid) over 60 min. Eluted peptides were directly electrosprayed into the mass spectrometer operated in positive mode and peptide analysis was performed using data-dependent acquisition of MS scan (m/z from 400 to 1800) followed by MS/MS scans. The MS/MS data were extracted using Analyst software version 1.5.1 (Applied Biosystems). The detailed analysis was performed as described previously (Chakraborty et al., 2013). For 4800 MALDI-TOF/TOF (Applied Biosystems) analysis, α-cyano-4-hydroxycinnamic acid (CHCA) matrix was prepared at one-half saturation in acetonitrile/water 1/1 (v/v) acidified with 0.1% TFA. A 1 μL aliquot of each sample was mixed with an equal volume of matrix solution. The mixture was immediately spotted onto the MALDI target plate and allowed to dry at room temperature. The reflected spectra were obtained over a mass range of 850 to 4000 Da. The spectra of 100 laser shots were summed to generate a PMF for each protein digest. Suitable precursors for MS/MS sequencing analyses were selected, and fragmentation was carried out using collision-induced dissociation (CID; atmospheric gas was used) in 1 kV ion reflector mode and precursor mass windows of +5 Da. The plate model and default calibration were optimized for processing the MS/MS spectra.

### Database searching, protein identification and expression clustering

The m/z spectra were searched against SGN Tomato database ITAG 2.3 release (34,727 sequences, 11956401 residues) available at http://solgenomics.net/organism/Solanum_lycopersicum/genome using the Mascot v.2.1 (http://www.matrixscience.com). For LC-MS/MS analysis, peak lists were searched against the combined target database using the MASCOT v.2.1. The database search criteria were: peptide tolerance, ±1.2 Da; MS/MS tolerance, ±0.6 Da; peptide charge +1 +2 or +3; maximum allowed missed cleavage, 1; fixed modification, cysteine carbamidomethylation; variable modifications, methionine oxidation; instrument type, ESI-TRAP. The score threshold to achieve *p* < 0.05 is set by Mascot algorithm and is based on the size of the database. For MALDI-TOF/TOF analysis, using GPS explorer v 3.6 (Applied Biosystems) against the aforesaid combined database. Search parameters were as follows: trypsin with one missed cleavage; fixed modification, cysteine carbamidomethylation; and variable modification, methionine oxidation; MS peak filtering: monoisotopic, minimum S/N 10, mass tolerance ± 100 ppm; MS/MS peak filtering: monoisotopic, minimum S/N = 3, MS/MS fragment tolerance ±0.4 Da. Proteins with C.I. % > 95% were considered as a positive identification and were also evaluated on the basis of number of peptides matched, MOWSE score, quality of the peptide maps, % coverage of the matched protein, besides similarity of theoretical and experimental protein molecular masses. For proteins identified by only one peptide having a score >40, the peptide sequence was checked manually. The amino acid sequences of identified proteins were enlisted in Supplementary Table S4. The significance threshold was set to *p* < 0.05 and false discovery rate (FDR) < 0.05 for the Mascot search. The protein functions were assigned using a protein function database, Pfam or InterPro. The identified proteins were divided into functional classes according to gene ontology (GO) and literature. BLASTP search of identified protein sequences was performed through Blast2GO (Conesa et al., 2005) against Uniprot protein database with a minimum expectation value of 1 × 10^−3^. Annotations were retrieved with default parameters: pre-eValue-Hit-Filter at 1 × 10^−6^, cut-off was set at 55 and GO weight at 5. Self-organizing tree algorithm (SOTA) clustering was performed on the log-transformed fold induction using Multi Experiment Viewer (MeV) software (Saeed et al., 2003). The clustering was done with the pearson correlation as distance with 10 cycles and a maximum cell diversity of 0.8 (Romijn et al., 2005). *p* < 0.05 were considered statistically significant.

### Primer design and qRT-PCR analysis

Seven key post- harvest storage responsive proteins were selected from the wild-type and E8.2-OXDC fruit proteome for a follow-up study of gene expression by qRT-PCR analysis. The nucleotide sequences of the corresponding proteins ware obtained by performing a TBLASTN search in SGN tomato database. For each candidate gene, primers were designed using Primer Express v3.0 software (Applied Biosystems). Primer sequences are listed in Supplementary Table S5.

Total RNA from 0 to120 h tomato fruits under storage were isolated using RNeasy Plant Mini Kit (Qiagen). The reverse transcription was carried out with 2 μg of RNA using SuperScript VILO cDNA Synthesis Kit (Invitrogen). The qRT-PCR was performed in three biological and three technical replicates with the ABI PRISM 7700 Sequence Detection System (Applied Biosystems) using SYBR Green PCR Master Mix and gene-specific primer pairs in a final volume of 20 μl, including cDNA template. 18 s rRNA is used as endogenous control for normalizing the qRT-PCR data and relative quantification (2^−ΔΔCt^). Expression ratios of mRNA transcripts at 24, 48, 72, 96, and 120 h relative to control (0 h) were calculated and statistically tested.

### Protein correlation network

The protein expression data from wild-type and E8.2-OXDC fruits across storage time points were used to calculate pearson correlation coefficient (PCC). The PCC was used as similarity index between any two proteins. Correlation pairs were considered significant with similarity strength more than 0.9 and selected as candidate interactions. All self-correlations and duplicate protein entries were removed to maintain the data non-redundancy. Cytoscape software (version 3.2.1) (Shannon et al., 2003) was used to visualize the resultant network and analyze network properties such as the average node degree, clustering coefficient, and network density.

### Statistical analysis

The statistical significance of each of the time point dataset on the normalized spot volumes were evaluated by One-way ANOVA (*p* < 0.05) with Bonferroni *post-hoc* correction using MeV (Saeed et al., 2003). Principal component analysis was performed using PCA functions of the XLSTAT Pro Version 2012.4.03 (www.xlstat.com) software. Values of all parameters analyzed are of three biological replicates per sample.

## Results

### Comparative proteome analyses during post-harvest storage in wild-type and E8.2-OXDC fruits

**Figure 1:**
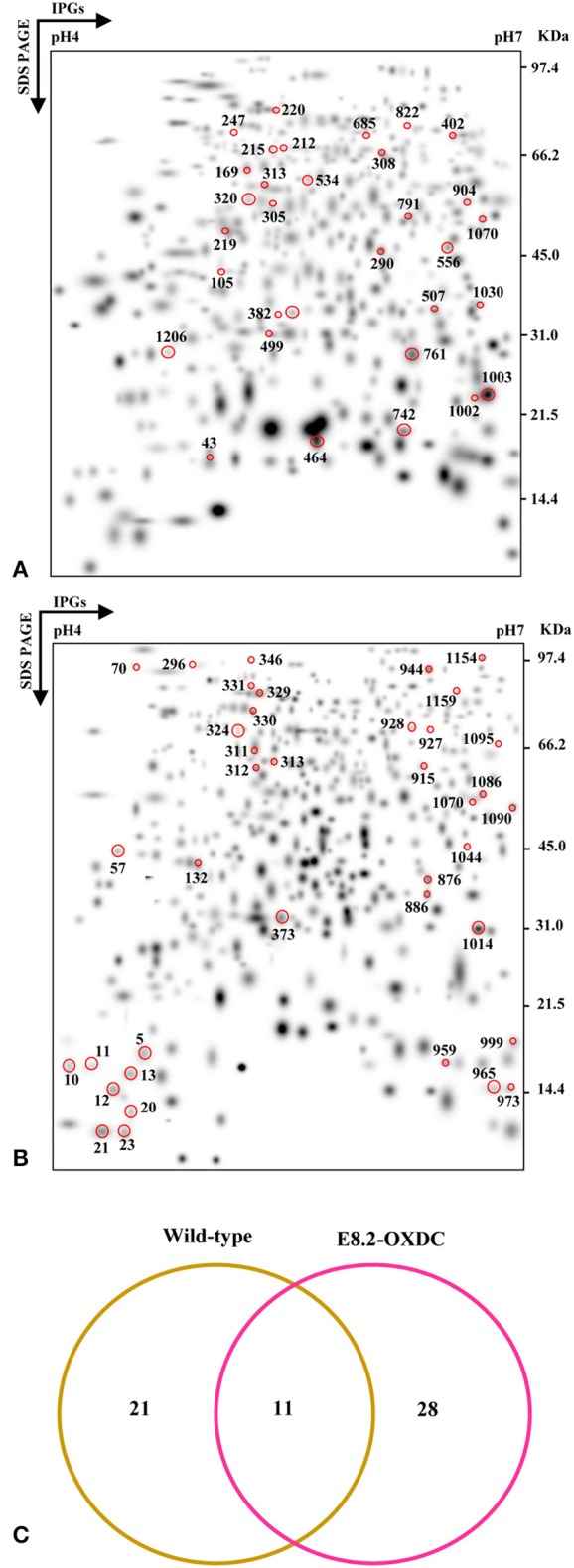
**Comparative proteomic analysis of wild-type and E8.2-OXDC tomato fruits during storage**. Three replicate 2-DE gels from Wild-type **(A)** and E8.2-OXDC **(B)** tomato were combined computationally using BIO-RAD PDQuest software (version 7.2.0) to generate standard gels. **(C)** Venn diagram showing the specific and overlapping protein spots from the wild-type and E8.2-OXDC fruits.

The temporal proteomic changes were monitored using high resolution 2-DE of total proteins from wild-type and E8.2-OXDC fruits during post-harvest storage till 120 h. A second level matchset was created that showed 224 spots and 220 spots (log2 > 1.32, *p* < 0.05) showing differential abundance at one or more time points during storage in wild-type and E8.2-OXDC fruits, respectively (Figures 1A,B). MS/MS analysis led to the identification of 32 Storage Related Proteins (SRPs) and 39 OXDC Regulated Storage Related Proteins (ORSRPs) in wild-type and E8.2-OXDC fruits, respectively. ANOVA analysis indicated a significant difference in proteins during storage at different time points (ANOVA, *p* < 0.05). Of the 32 identified SRPs in wild-type fruits, 17 spots were upregulated, 5 spots showed downregulation while 10 exhibited mixed pattern of temporal abundance during storage (Supplementary Tables S1, S3). Further, 39 ORSRPs identified from E8.2-OXDC fruits revealed that 18 spots were upregulated, 13 spots showed downregulation while 8 exhibited mixed pattern of temporal abundance during storage (Supplementary Tables S2, S3).

**Figure 2:**
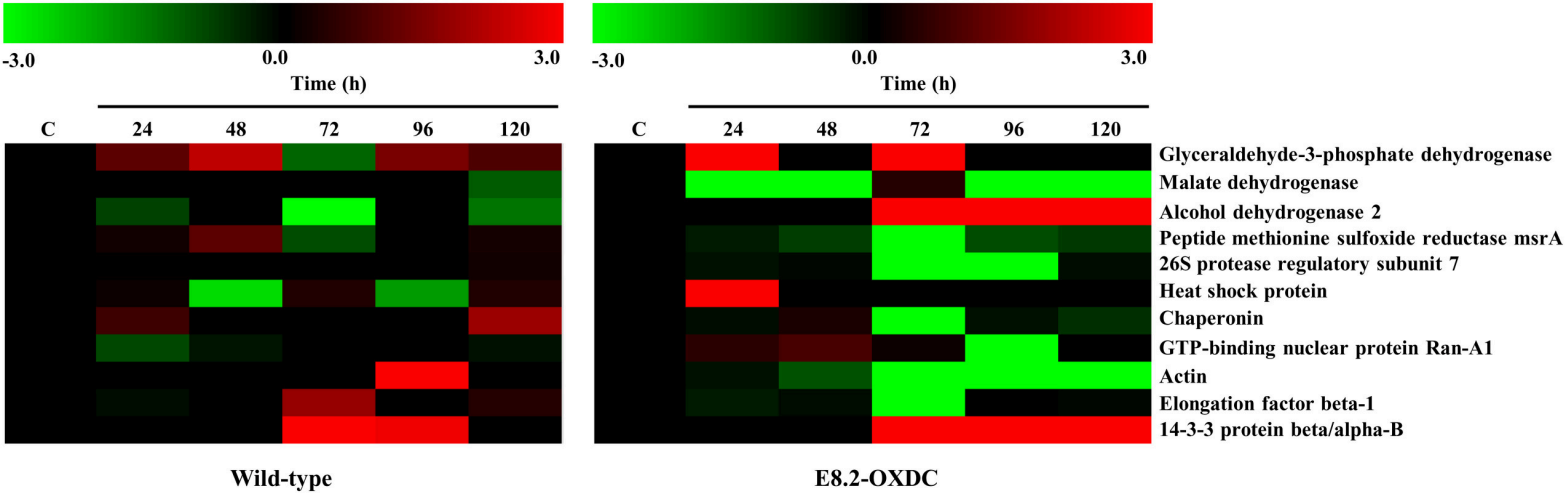
**Heat map of identified proteins found common between wild-type and E8.2-OXDC fruits**. The red color represents relatively high expression, and green color represents relatively low expression level.

The abundance pattern and function of 11 Common Storage Related Proteins (CSRPs) were investigated in wild-type and E8.2-OXDC fruits during storage condition (Figure 1C). CSRPs were subjected to clustering using their abundance ratios. In wild-type fruits, out of 4 increased proteins, one each was found to belong in the category of transport, transcription regulation, signaling and protein folding degradation, respectively; whereas the 2 decreased proteins were predominantly related to metabolism and protein folding and degradation. In E8.2-OXDC fruits, 3 proteins were increased that included 2 metabolism-related proteins, whereas the 4 decreased proteins were mainly categorized in protein folding degradation and signaling functional groups (Figure 2).

To study the correlated expression abundance pattern of SRPs and ORSRPs in wild-type and E8.2-OXDC fruits during storage, SOTA clustering was performed. The analysis yielded 11 expression clusters (Supplementary Figure S1). The most abundant groups detected in wild-type fruits, Clusters 4 and 10 were found to be dominated by metabolic, protein turnover and redox related proteins. Cluster 3 included proteins that were downregulated during storage and involved in metabolism and signaling. Cluster 1 and 2 showed upregulation during initial stage of storage. Further, proteins identified in E8.2-OXDC fruits under storage were examined by clustering analysis based on their abundance ratios. The most abundant groups were Clusters 7 and 1 associated with metabolism, stress response and redox related proteins. While Cluster 5 and cluster 9 consisted of proteins downregulated during storage. Proteins in Cluster 3, 8, and 11 showed upregulation in storage related to metabolic activities.

### Functional distribution and dynamics of SRPs and ORSRPs

**Figure 3:**
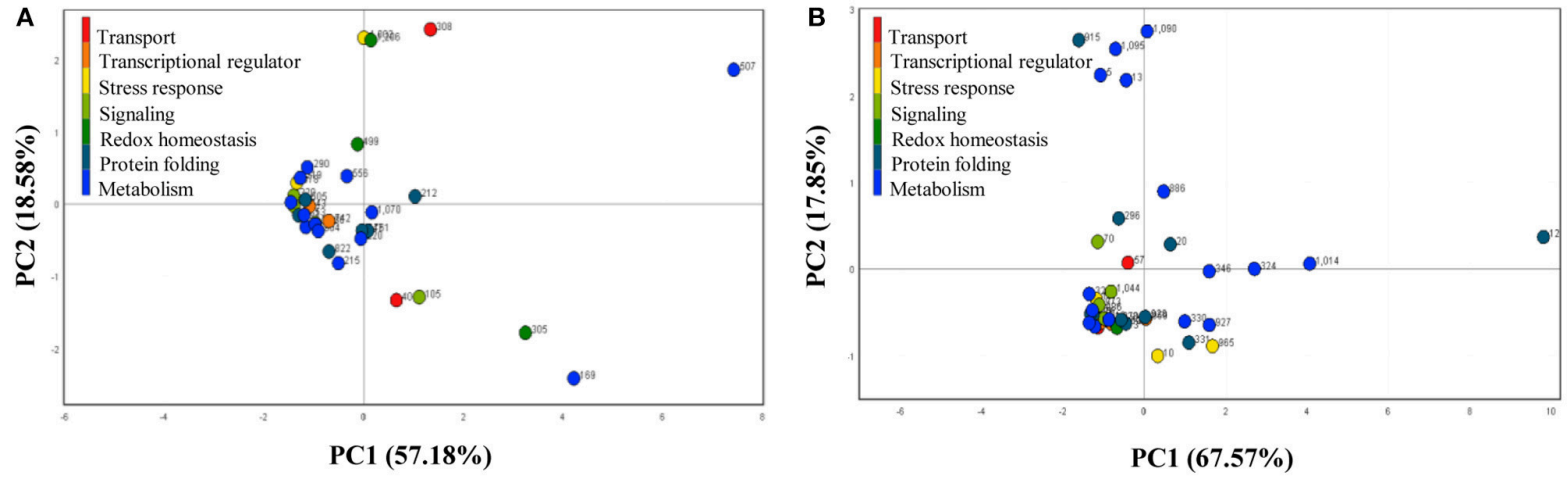
**Principal component analysis of the identified proteins from (A) wild-type and (B) E8.2-OXDC fruits**. X-axis represents principal component 1 and Y-axis represents principal component 2.

To understand the temporal proteomic dataset with exploratory statistic in wild-type and E8.2-OXDC fruits during storage, differentially abundant identified spots from two counterparts were subjected to PCA to interpret proteome data for significant variability and relevance with respect to time kinetics. Scree plot of time kinetics data during fruit storage in wild-type and E8.2-OXDC reflected variation through four principal components (Figure 3). Biplot analysis on plotting PC1 (57.18%) against PC2 (18.58%) in wild type and PC1 (67.57%) against PC2 (17.87%) in E8.2-OXDC fruits indicated that concerted action of SRPs and ORSRPs contribute to multifactorial responses through translational reprogramming during post-harvest storage. Hence, the PCA analysis and biplot revealed a time dependent storage related protein abundance dynamics. Proteins spots that showed significant difference in abundance may be considered as potential biomarkers associated with low oxalate mediated effect of storage on various sensorial attributes.

**Figure 4:**
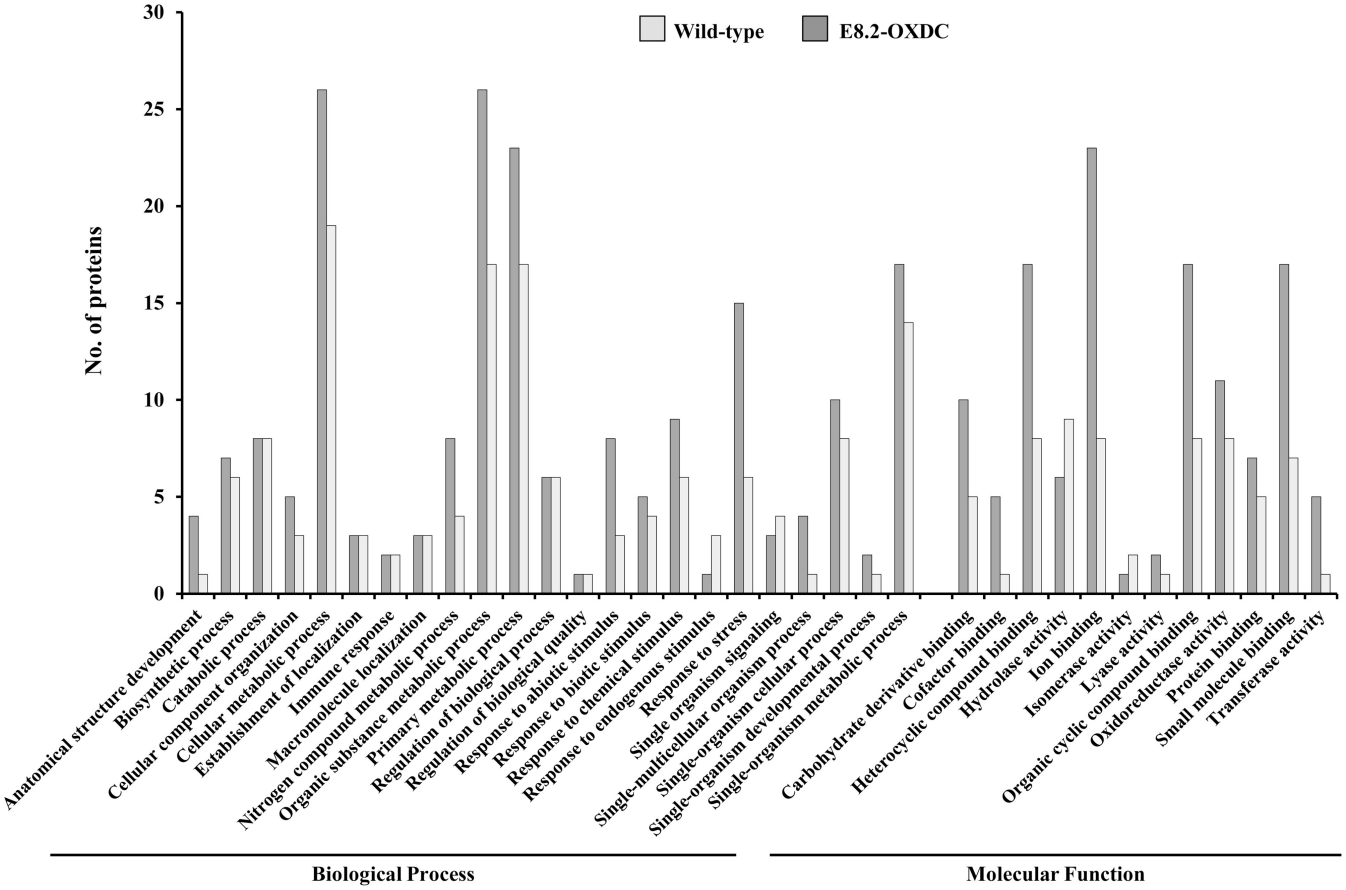
**Functional enrichment of the common proteins between wild-type and E8.2-OXDC fruit**. Distribution of common protein between wild-type and E8.2-OXDC fruit according to GO functional category (biological process and molecular function) by Blast2GO. The X-axis exhibits GO terms and the Y-axis shows number of proteins.

To achieve a comprehensive overview of comparative protein profile, identified SRPs, ORSRPs and CSRPs were sorted into seven functional categories (Supplementary Figure S2, Supplementary Tables S1–S3). The abundance of 32 SRPs identified in wild-type fruits under storage were functionally categorized as being predominantly involved in metabolism (34%), protein folding modification and degradation (19%), and redox homeostasis and signaling 13% each. The differentially abundant 39 ORSRPs detected in E8.2-OXDC fruits were categorized in metabolism (38%), protein folding modification and degradation (26%), and signaling 10% (Supplementary Figure S2). The number of proteins related to redox homeostasis and stress response markedly differed during storage compared to the other functional categories. Metabolism, protein folding, degradation and signaling-related proteins were majorly deregulated in both wild-type and E8.2-OXDC fruits. Overall the identified proteins were associated with cellular and organic substance metabolic processes that relate to ion binding, small molecule binding and oxidoreductase activity (Figure 4).

### Correlation network of differentially abundant SRPs and ORSRPs

**Figure 5:**
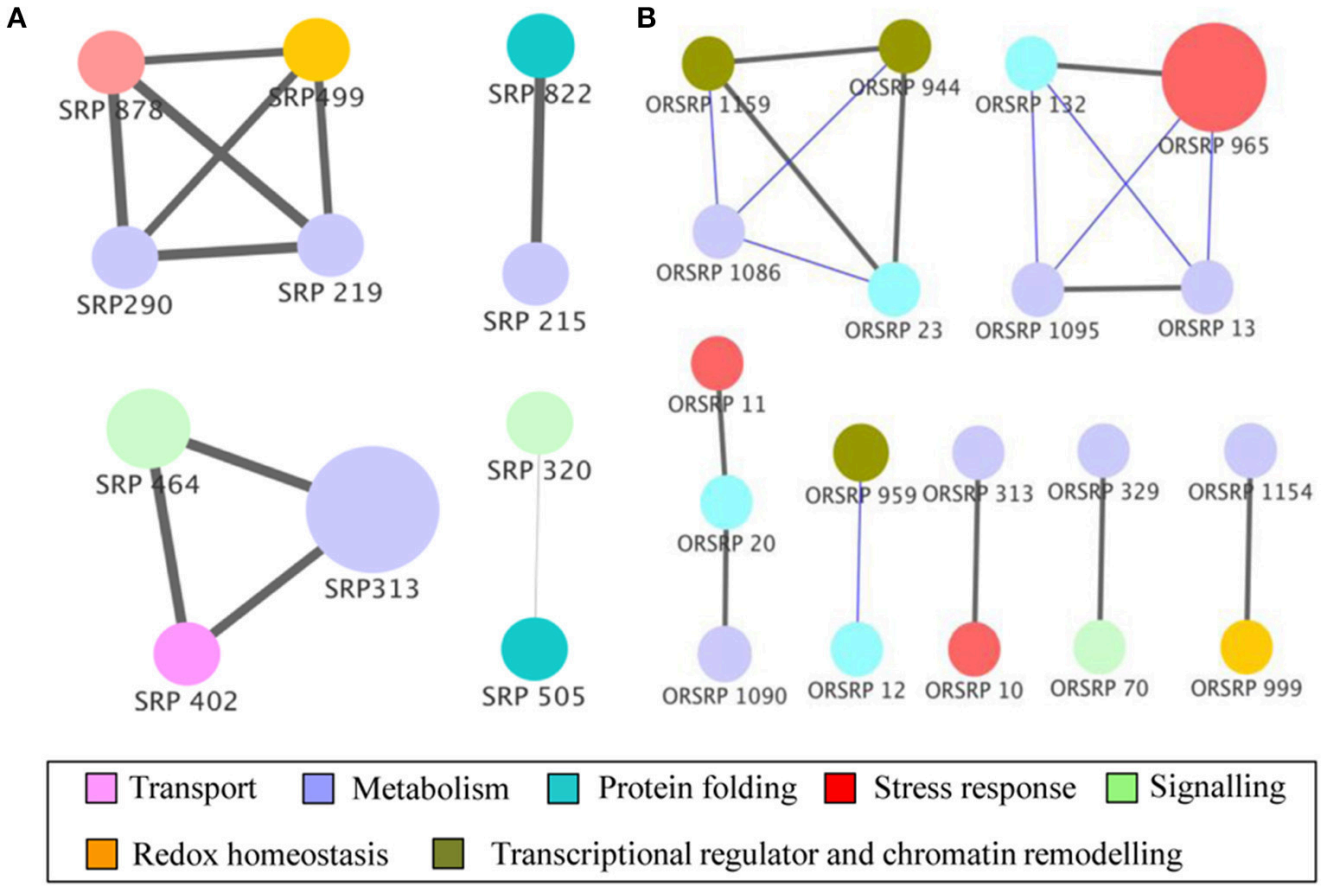
**Functional correlation network of identified proteins**. Proteins from wild-type **(A)** and E8.2-OXDC **(B)** sets were subjected to partial Pearson correlation analysis visualized in Cytoscape. Node colors represent functional categories while node size represents ANOVA *p*-values. Gray and blue edges correspond to positive and negative partial correlation, respectively. Edge width corresponds to strength of correlation.

To elucidate the post-harvest storage attributes in responses to oxalate downregulation, we performed a correlation analysis to assign significance level to the proteins. We obtained a protein correlation network of 11 nodes and 10 edges in wild-type. The network at an alpha value of 0.01 contains two modules (M) and one small correlation groups (SC) of just 2 proteins. Further, we succeeded in extracting functional relationships from the concatenated data sets of 20 nodes and 10 edges based on the storage effect in E8.2-OXDC tomato. Network contained one module and six correlation groups (SC) of 2 to 3 proteins (Figure 5).

The post-harvest storage network of wild-type was composed of module 1(M1) of four proteins, module 2 (M2) of 3 proteins and one small correlation group (SC1) of two proteins. Proteins in M1 are related to sensorial attributes, biogenesis of fatty acids and protein related to redox homeostasis involving 6-phosphogluconolactonase, 2-oxoglutarate-dependent dioxygenase and glutathione transferase. Further, proteins in M2 are linked to transport, protein homeostasis and acid balance during storage containing v-type ATP synthase beta chain, F-box protein PP2-B1 and acid beta-fructofuranosidase. Interestingly, network comprised of small correlation group (SC1) with 26S protease regulatory subunit 7 and acetyl esterase, indicating its role in storage and biogenesis with assembly of fatty acid components (Figure 5A).

The correlation network related to E8.2-OXDC during storage comprised of single module of four proteins consists of T-complex protein eta subunit, fructose-bisphosphate aldolase, asparaginyl-tRNA synthetase, and aspartic proteinase. The cascade in M3 contains proteins involved in carbohydrate metabolism, osmotic regulation, and protein modification. Proteins found in SC2-SC7 were related to fruit quality maintenance. Interaction between biological machineries may affect storage attributes resulting in potential physiological state. Altogether this data demonstrates that status of protein during feedback regulation can functionally link translational changes to storage (Figure 5B).

### Expression levels of key genes

**Figure 6:**
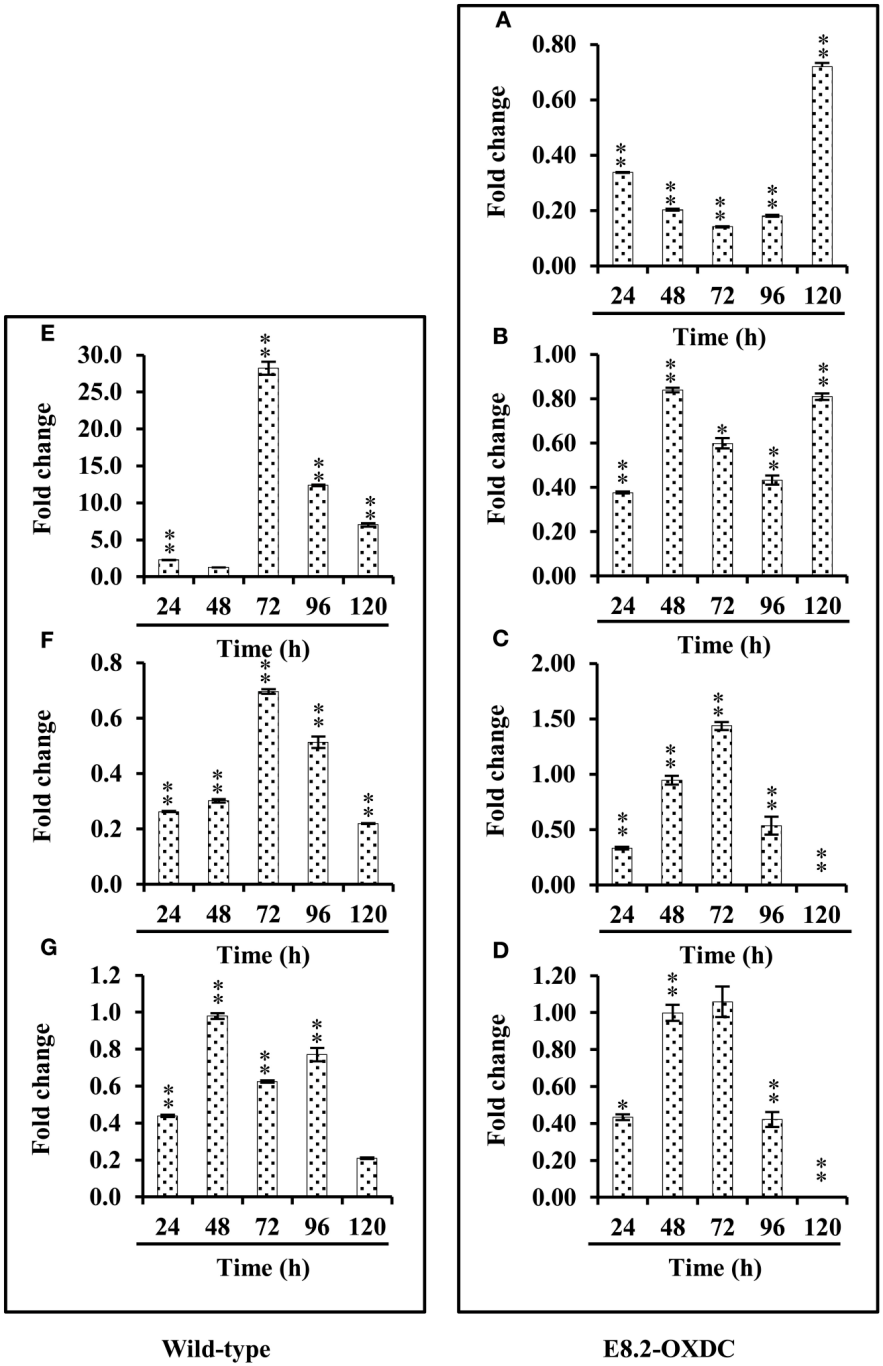
**Relative quantification (RQ) of mRNA levels**. Candidate genes from wild-type and E8.2-OXDC fruits stored at 24, 48, 72, 96, 120 h were analyzed by qRT-PCR. Expression changes were analyzed by ANOVA and vertical bars indicates SE. Significantly up- or down- regulated genes are indicated by ^*^ for *p* < 0.05 (Tukey *post-hoc test*). **(A)** Polygalacturonase A **(B)** 5-methyltetrahydropteroyltriglutamate-homocysteine methyltransferase, **(C)** Alcohol dehydrogenase 2, **(D)** GTP-binding nuclear protein Ran-A1, **(E)** 14-3-3 **(F)** Adenosylhomocysteinase, **(G)** Peptide methionine sulfoxide reductase msrA.

Based on the proteomic results, key enzymes involved in storage related pathways, that showed changed abundances in wild-type and E8.2-OXDC fruits were selected for mRNA expression analysis. The qRT-PCR results indicated that the mRNA expression level of SRPs exclusive in wild type namely, 14-3-3 and adenosylhomocysteinase were down-regulated from 72 to 120 h during storage and that of peptide methionine sulfoxide reductase showed slightly increased expression in accordance with the proteome study. Correlation between gene expression levels and protein abundance data of exclusive ORSRPs revealed that genes encoding polygalactouranase A, and 5-methyltetrahydropteroyltriglutamatehomocysteinemethyl transferase involved in cell wall biogenesis associated with fruit firmness showed increased expression during later phase of storage. However, alcohol dehydrogenase 2 and GTP-binding nuclear protein Ran-A1 showed differential expression during storage (Figure 6).

### Investigation of physiochemical changes in wild-type and low oxalate tomato

**Figure 7:**
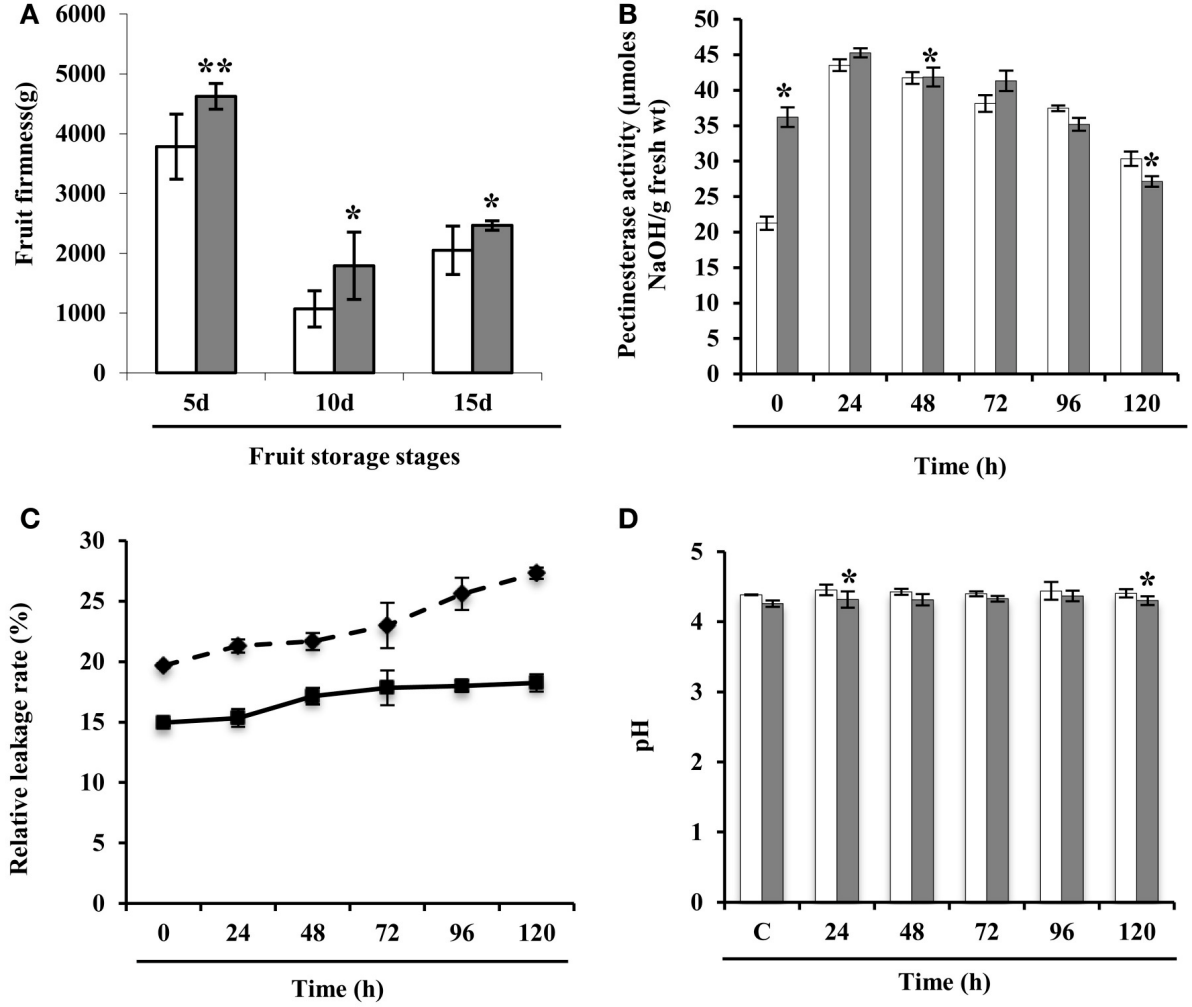
**Physio-chemical analysis of wild-type and E8.2-OXDC tomato fruits**. **(A)** Firmness analysis of fruits harvested at 5, 10, and 15 days after the first color change (the breaker stage). Firmness was determined using a texture analyzer. Values shown are the mean ± SD from 8 fruits **(B)** Pectinesterase activity **(C)** Relative leakage rate and **(D)** pH change. Data are means of four replicates ± SE. White bar represent wild-type fruits and black bar represent E8.2-OXDC fruits. Asterisk indicates significant differences among time points according to Duncan's multiple range tests (*p* < 0.05).

Next, we investigated the underlying mechanism that might be the result of OA downregulation during storage. To quantify texture, we analyzed the firmness of the wild-type and E8.2-OXDC fruits, which revealed enhancement of firmness in the transgenic fruit. Ten days after the storage, E8.2-OXDC fruits were ≈2.0-fold firmer than wild-type fruits and showed no signs of deterioration up to 15 days (Figure 7A). Firmness is linked to cell wall properties and composition of structural polysaccharides, particularly pectin allows primary cell wall strengthening. To understand the wall properties related to fruit firmness, we measured the activity of pectinesterase, a pectin modifying/degrading enzyme in wild type and E8.2-OXDC tomato till 120 h of storage. Our study showed increased activity of pectinesterase in E8.2-OXDC fruits till 24 h post-harvest storage in comparison to the wild-type fruit. However, during 96–120 h of post-harvest storage wild-type fruits exhibited decreased pectinesterase activity compared to that of E8.2-OXDC fruits (Figure 7B). We also evaluated the membrane permeability which was expressed as relative leakage rate. Although the relative leakage rate in E8.2-OXDC fruit marginally decreased with respect to wild-type fruits during storage, increase in relative leakage was observed with increasing storage time in both wild-type and E8.2-OXDC fruits (*p* < 0.05) (Figure 7C). In addition, there was no significant difference in pH between E8.2-OXDC fruit and wild-type, and pH was in the range of 4.28–4.37 during storage (Figure 7D). Furthermore, we assessed the fruit quality by determining the levels of bioactive compounds, such as lycopene and organic acids (Supplementary Figure S3). Lycopene content was marginally increased in E8.2-OXDC fruits compared to wild-type till 120 h post-harvest storage. Biochemical analysis revealed substantial reduction in oxalic acid (up to 50–85%) in E8.2-OXDC fruit as compared to wild-type. To investigate whether this decrease in oxalic acid levels leads to corresponding changes in the abundance of other organic acids, we measured malic acid and citric acid in wild type and E8.2-OXDC fruit during post-harvest storage. We observed that citric acid content increased in early stages and maintained a steady state levels thereafter till 120 h during storage in E8.2-OXDC fruit. However, malic acid showed marginal downregulation and subsequent sustenance at steady state level at later stage during storage.

## Discussion

**Figure 8:**
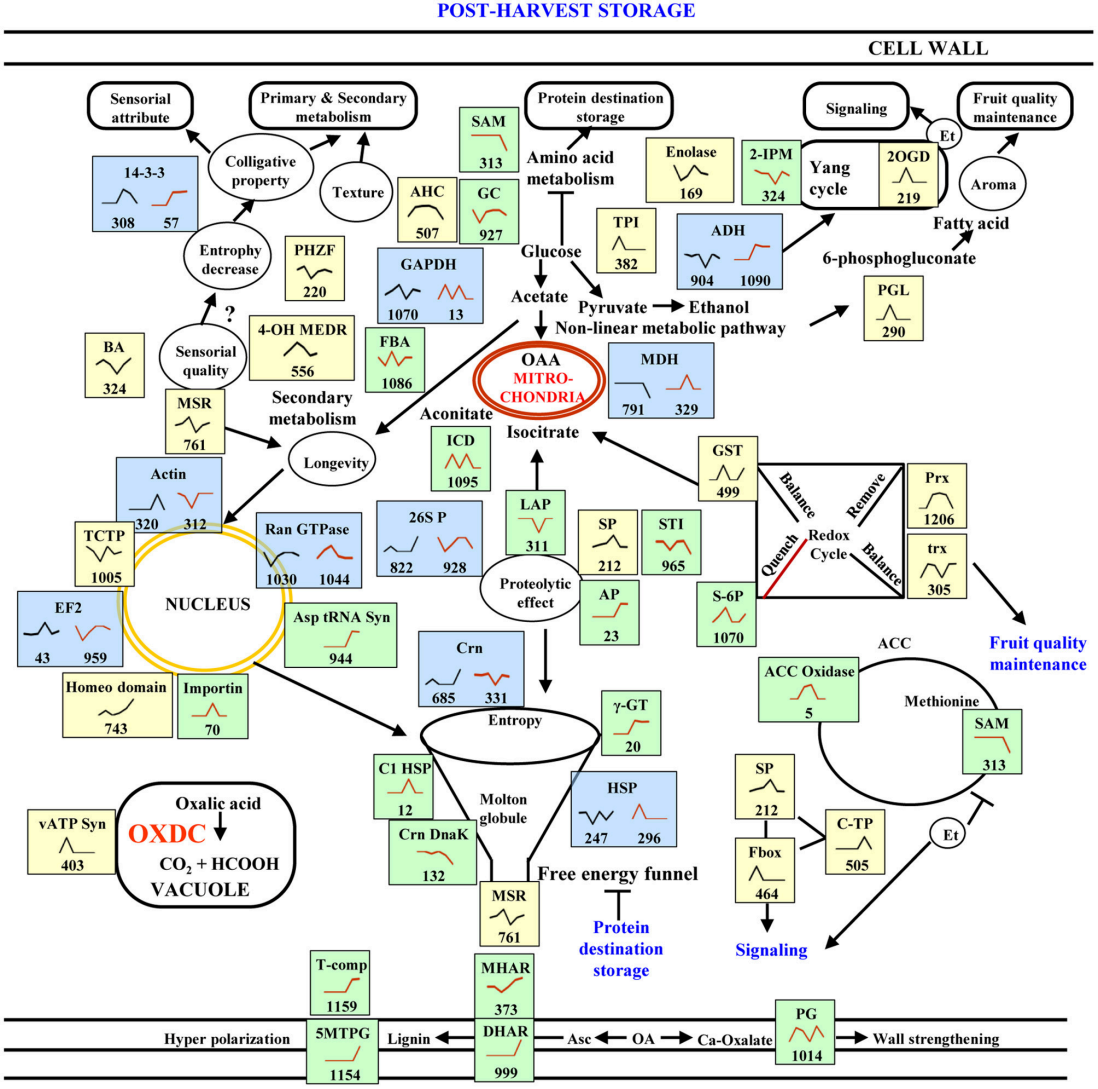
**A model summarizing the cellular pathways associated with storage in wild-type and E8.2-OXDC fruits**. Proteins identified in this study are indicated in blue box for common proteins, yellow box indicate proteins exclusive to wild-type fruit and green box represents proteins exclusive to E8.2-OXDC fruits. Graphs are representative of abundance patterns of individual proteins and the number given below each graph indicates the protein identification number. Line graphs in black represents abundance pattern of wild-type fruits and brown line graphs represent abundance pattern of E8.2-OXDC. The abbreviations are: TPI, triose phosphate isomerase; SAM, S- adenosylmethionine; AP, ascorbate peroxidase; GC, glutamate carboxylase; AHC, adenosyl homocysteinase; C-I HSP, class- I heat shock protein; HSP, heat shock protein; GST, glutathione S- transferase; ACC oxidase; 1-aminocyclopropane-1-carboxylate oxidase-like protein; 4-OH MEDR, 4-hydroxy-3-methylbut-2-enyl diphosphate reductase; TCTP, translationally controlled tumor protein; GAPDH, glyceraldehyde 3-phosphate dehydrogenase; PHZF, Phenazine biosynthesis protein PhzF family; MDH, malate dehydrogenase; 2-IPM, 2-isopropyl malate synthase; BA, betaine aldehyde dehydrogenase; PGD, phosphoglycerate dehydrogenase; PGK, phosphoglycerate kinase; Prx, peroxiredoxin; MSR, methionine sulfoxide reductase; EF2, elongation factor 2; vATP Syn, v-type ATP synthase; LAP, leucyl amino peptidase; FBA, fructose bisphosphate aldolase; Crn, chaperonin; 5 MTPG, 5-methyltetrahydropteroyltriglutamate– homocysteine methyltransferase; MHAR, Monodehydroascorbate reductase; DHAR, Dehydroascorbate reductase; PG, polygalacturonase; c-TP, carboxy terminal protease; SP, Serine carboxypeptidase; S-6P, sorbitol 6-phosphate dehydrogenase.

Post-harvest storage of fruit involves a series of physiological, biochemical, and organoleptic changes accompanied by carbohydrate build-up, acid reduction, and carotenoid accumulation (Talon and Gmitter, 2008). Our temporal analyses of protein abundance in E8.2-OXDC tomato fruits revealed distinct abundance patterns compared to wild-type fruits during post-harvest storage. Further, we investigated the differences in the accumulation of proteins at similar time point between wild type and E8.2-OXDC fruits that affect the storage quality. We next employed a systems approach to identify regulatory proteins that impact on sensorial attributes, metabolism, storage, signaling and fruit quality maintenance. This work aims to precisely correlate protein variations with fruit storage as a consequence of oxalate downregulation. A model representing the regulatory and functional network of low oxalate responsive proteins in fruit quality and post-harvest storage is depicted in Figure 8.

### Fruit metabolism is linked to sensorial attributes during storage

Among the 23 differentially abundant proteins related to metabolism, 8 were found exclusively in wild-type fruit, 12 in E8.2-OXDC fruit and 3 were common to both during storage. An analysis of their biological process and molecular function categories showed that glycolytic pathway, TCA cycle, yang cycle, amino acid metabolism and phenylpropanoid pathway are pivotal to storage related processes. Color and aroma are two major sensorial attributes of tomato fruits. Some of the enzymes related to glycolytic and TCA cycles, namely alcohol dehydrogenase (ADH) and malate dehydrogenase (MDH) plays crucial role in starch metabolism, accumulation of soluble solid content and production of aroma during post-harvest storage (Manríquez et al., 2006; Centeno et al., 2011). The quantitative proteomics data indicate ADH and MDH as CSRPs that showed variable abundance pattern in wild-type and E8.2-OXDC fruits. MDH (SRP-791, ORSRP-329) was not altered till 48 h either in wild-type or E8.2-OXDC fruit after harvest. Thus, ADH had antagonistic abundance pattern with more than 10-fold upregulation in E8.2-OXDC fruit indicating that transgenic fruits might exhibit improved fruit quality compared to wild-type. Whereas, ADH was significantly downregulated in wild-type fruit and showed upregulation from 72 to 120 h in E8.2-OXDC fruit after harvest. At transcript level exclusively in E8.2-OXDC fruit, ADH showed increased expression (1.5-fold) at 72 h in accordance with protein profile. Therefore, both MDH and ADH might be involved in the biosynthetic pathway leading to esters (such as hexanal) formation and improved aroma in E8.2-OXDC tomato fruit. Further protein profiling showed three proteins that had either higher (one protein) or lower (two proteins) abundance in E8.2-OXDC fruit were related to glycolysis. Of these, 2,3-bisphosphoglycerate phosphoglycerate mutase (ORSRP-330) and fructose-bisphosphate aldolase (ORSRP-1086) were downregulated at 24 h while glyceraldehyde 3-phosphate dehydrogenase (GAPDH) (ORSRP-13) showed upregulation at 24 and 72 h during storage modulating organic acid pool due to low oxalate levels. Furthermore, two enzymes related to glycolysis were detected exclusively in wild-type tomato, namely triosephosphate isomerase (TPI; SRP-382) and beta-fructofuranosidase (FF; SRP-313). TPI and FF showed increased abundance at 24 h implying that metabolite partitioning is influenced by the stage and organoleptic properties of the fruit. Another protein identified exclusively in wild-type fruit was acetyl esterase (SRP-215), which showed down-regulation at 24 h and known to be involved in regulation of volatile constituent of fruit that constitute aroma and flavor implying thereby secondary metabolite pool was affected during storage in wild-type fruit (Goulet et al., 2012). Additionally, enzymes related to flavonoid, lycopene and ethylene biogenesis namely 2-oxoglutarate-dependent dioxygenase (SRP-219) and 4-hydroxy-3-methylbut-2-enyl diphosphate reductase (SRP-556) present exclusively in wild-type fruit showed increased abundance indicating that mevalonate pathway was deregulated during storage. Oxalate downregulation also favors marginally increased lycopene content in E8.2-OXDC fruits compared to wild-type improving fruit antioxidant status. We conclude that conversion of 6-carbon intermediate to 2-carbon intermediate by non-linear catabolic pathways was affected due to oxalate downregulation during post-harvest storage. We further hypothesized that decrease in levels of oxalic acid could have a direct bearing on the fruit intracellular acid pool by balancing ratio of malic acid and citric acid to maintain nutritional status of E8.2-OXDC fruit during storage.

Concerning amino acid metabolism, three proteins such as 2-isopropylmalate synthase 1 (IPMS; ORSRP-324), glutamate decarboxylase (ORSRP-927) and S-adenosylmethionine-dependent methyltransferase (SAM dependent methyl transferase; ORSRP-313) involved in methionine and hydrophobic amino acid metabolism showed downregulation in E8.2-OXDC fruit, particularly at 24 h and 120 h during storage. However, in wild-type fruit adenosylhomocysteinase (SRP-507), another methionine biosynthesis enzyme showed upregulation. These data suggests the antagonistic feedback regulation of methionine biosynthesis between wild-type and E8.2-OXDC fruit during storage. Previous studies have suggested that, glutamate decarboxylase (GAD) catalyzed conversion of glutamic acid to gamma aminobutryric acid (See review Bouché and Fromm, 2004). Furthermore, IPMS and SAM dependent methyl transferase involved in a variety of cellular processes; notably, a precursor of ethylene synthesis and of the phenylpropanoid constituents of the cell wall (Espartero et al., 1994). This study points toward the fact that downregulation of above mentioned enzymes might lead to the accumulation of amino acids and reduction of ethylene in E8.2-OXDC fruit. Adenosylhomocysteinase catalyze conversion of homocysteine to methionine which is an intermediate in the formation of ethylene implying thereby ethylene production is favored in wild-type fruit. Furthermore, in wild-type fruit, adenosylhomocysteinase (methionine biosynthesis enzyme) showed increased abundance from 24 to 96 h in proteomic analysis. At transcript level also adenosylhomocysteinase showed maximum increase at 72 h (0.7-fold).

The Pentose phosphate pathway (PPP) in plant is the major source of reduced nicotinamide adenine dinucleotide phosphate (NADPH) and ribulose 5-phosphate, which are involved in the synthesis of aromatic amino acids and fatty acids (Huang et al., 2003). 6-phosphogluconolactonase (SRP-290), an enzyme participating in PPP, increased at 48 h during post-harvest period in wild-type fruit, suggesting its role in the decrease of glucose and fructose maintaining turgor pressure. Further, isocitrate dehydrogenase, a TCA and PPP pathway enzyme known to provide metabolic signal that could be implicated in ammonia re-assimilation or in glutamic acid accumulation contribute to fruit flavor (Gallardo et al., 1995). Our results showed increased abundance of isocitrate dehydrogenase (ORSRP 1095) in E8.2-OXDC fruit at 24 h and 72 h during fruit storage. Thus, we conclude that, oxalate downregulation during storage regulate protein abundance of enzymes related to central metabolism and follow a pattern that is controlled by physiological and biochemical parameters of fruit.

Texture is one of the most important fruit quality attributes. Several factors contribute to the overall fruit texture, of which cell wall reorganization plays key role in regulating fruit shelf life (Vicente et al., 2007). Interestingly, some of the detected spots corresponded to proteins putatively involved in cell wall remodeling in E8.2-OXDC fruit. 5-methyltetrahydropteroyltriglutamate-homocysteine methyltransferase (ORSRP-1154) and NADP dependent sorbitol 6-phosphate dehydrogenase (ORSRP-1070) showed higher abundance particularly at 120 h, while polygalactouranase A exhibited increased abundance at 96 h of storage in E8.2-OXDC fruit. Notably, qRT-PCR analysis of 5-methyltetrahydropteroyltriglutamate-homocysteine methyltransferase revealed that both transcript and protein profile were similar; however, the magnitude of fold change was lower in the transcript profile (0.8-fold). Also, transcript level of polygalactouranase A exhibited increased expression at 120 h implying that wall reorganization is favored during storage in E8.2-OXDC fruit. This data indicate that oxalate downregulation leads to cell wall restructuring and tightening during storage. Besides, wild-type fruits displayed increased abundance of cell wall disassembly related enzymes like acid beta-fructofuranosidase (SRP-313) at 24 h during storage. Therefore, it is apparent that lignin biosynthesis is favored in E8.2-OXDC fruit due to oxalate downregulation, whereas depolymerization of structural polysaccharides is found in wild-type fruits. Firmness is linked to cell wall properties and composition of structural polysaccharides, particularly pectin that allows primary cell wall strengthening. During storage, it is targeted by cell wall modifying/degrading enzymes mainly pectinesterase. Pectinesterase either contributes to the cell wall stiffening by producing unesterified carboxyl groups that interact with calcium ions forming a pectate gel. The other being release of protons to stimulate activity of hydrolases leading to cell wall loosening. Here, we report that pectinesterase modulates catalysis of polygalactouronic acid for cell wall reinforcement in E8.2-OXDC fruit (Figure 7B). Our data clearly revealed that compared to wild-type fruits, E8.2-OXDC fruit had better quality attributes.

In previous reports ethylene had been shown to affect the activities of essential enzymes, such as 1-aminocyclopropane-1-carboxylate oxidase (ACC oxidase) involved in the volatile biosynthetic pathways and yang pathway (Zhu et al., 2005; Klee and Giovannoni, 2011). ACC oxidase, a rate limiting enzyme that maintain ethylene pool and liberation of volatile metabolites. We found increased abundance of ACC oxidase (ORSRP-5) at 48 and 72 h in E8.2-OXDC tomato fruits during storage. The qRT-PCR analysis also revealed that expression of ACC oxidase was significantly downregulated from 0 to 48 h followed by marginal increase till 120 h during storage indicating its role in volatile metabolite formation due to low oxalate levels that might contribute to fruit firmness and post-harvest storage.

### Proteomic changes are indicative of protein homeostasis mediated antioxidant balance during post-harvest storage

Post-harvest storage of fruits affects protein homeostasis machinery. In the present study, three protein folding and degradation related proteins were found to be common between wild-type and E8.2-OXDC fruit. Of these, CSRPs namely heat shock protein (SRP-247; ORSRP-296) and chaperonin (SRP-685; ORSRP-331) showed significant expression pattern difference between wild-type and E8.2-OXDC fruit during storage. Above-mentioned CSRPs exert protective effect by refolding damaged proteins, and renaturing aggregated proteins (Boston et al., 1996). The heat shock proteins are known to remodel or disassemble protein complexes using ATP. In the present study, class I heat shock protein (ORSRP-12) was significantly upregulated, while class IV heat shock protein (ORSRP-915) showed downregulation in E8.2-OXDC fruit. Protein degradation plays a pivotal role in sustaining cellular process by removing misfolded proteins and controlling the level of certain regulatory points. Leucyl aminopeptidase (ORSRP-311), serine carboxypeptidase K10B2.2 (SRP-212), carboxyl-terminal proteinase (SRP-505), and aspartic protease (ORSRP-23) are known to be energy-dependent, hormone regulator and unfold protein substrates to translocate misfolded proteins into the degradation chamber. Also, they play vital role in the protein quality control (Sanchez-Bel et al., 2012). Therefore, we speculate that oxalate downregulation and storage condition both might govern protein homeostasis. The abundance pattern of peptide methionine sulfoxide reductase (SRP-761; ORSRP-876) and 26S protease regulatory subunit 7 (SRP-822; ORSRP- 928) showed antagonistic behavior due to utilization of distinct protein for folding/degradation in wild-type and E8.2-OXDC tomato fruit during storage. This data revealed that the proteases and peptidases showed distinct pattern of abundance suggesting that protein degradation machinery is specific in regulating protein homeostasis during storage in wild-type and E8.2-OXDC fruits.

Antioxidants modulate the steady-state concentrations of ROS, regulate protein homeostasis and associated with sensorial attributes (Mittler et al., 2004). Antioxidant enzymes include peroxiredoxins (PRXs) and glutathione S transferase (GST) of the ascorbate–glutathione pathway associated with regeneration of monodehydroascorbate reductase (MR) and dehydroascorbate reductase (DR). We report that MR (ORSRP-373) and DR (ORSRP-999) showed increased abundance at 120 h in E8.2-OXDC fruit during storage. The observation reflects the fact that increase in ascorbate pool might lead to increased cell wall strength, better texture and color of transgenic fruit due to oxalate downregulation. Moreover, in wild-type fruit also some of the antioxidant enzymes, namely peroxiredoxin (SRP-1206) and glutathione transferase (SRP-499) showed increased abundance particularly at 48 h revealing that ascorbate–glutathione pool is affected during storage and is not dependent on oxalic acid concentration. Antioxidant potential seems to have close link to cellular trafficking. 14-3-3 proteins (SRP-308; ORSRP-57) function as regulator of important biological processes, such as metabolism, transcription, and organellar protein trafficking. It appeared as common protein and depicted synergistic protein pattern with increased abundance at 72 and 96 h in both wild type and E8.2-OXDC fruits during storage. The qRT-PCR analysis also revealed increased expression at 72 h correlating with proteome data. This suggests that 14-3-3 might function as turgor pressure regulator modulating cellular trafficking and water potential of cell during storage.

### Fruit quality maintenance and cytoskeleton dynamics during storage

Acclimatization, maintenance and exhaustion are three main aspects of post-harvest storage. Maintenance of fruit involves cytoskeleton reorganization and accumulation of solutes. Our study reports interplay of importin (ORSRP-70), GTP-binding nuclear protein Ran-A1(SRP-1030; ORSRP-1044), actin (SRP-320; ORSRP-312) nucleoside diphosphate kinase (ORSRP-973) and T-complex protein eta subunit (ORSRP-1159). We showed increased abundance of these proteins particularly at 72 and 96 h during storage. However, actin exhibited antagonistic abundance pattern with differential regulation in E8.2-OXDC fruits compared to wild-type reflecting that cytoskeleton components were restructured to modulate cellular turgor improving fruit quality attribute in oxalate down-regulated fruit. GTP-binding nuclear protein Ran-A1 also showed increased abundance at 24 h in wild-type and 96 h in E8.2 OXDC fruits, indicating that interplay of G-protein signaling components could fulfill fundamental role of modulating the low oxalate-mediated cellular responses in E8.2-OXDC fruits. Also, RNA expression revealed that GTP-binding nuclear protein Ran-A1 showed increased levels at 72 h during storage. Earlier it was reported that nucleoside diphosphate kinase is involved in macromolecule formation (De la Rosa et al., 1995). Moreover, role of importin and actin in cellular reorganization is well postulated (Mariot et al., 2015). Interestingly, function of T-complex protein eta subunit and GTP-binding nuclear protein Ran-A1 is still not known in fruit maintenance. Furthermore, v-type ATP synthase beta chain (SRP-402) acts an important regulator of membrane trafficking (Schumacher and Krebs, 2010). The high abundance of these proteins involved in energy metabolism and transport as well as in maintaining redox homeostasis postulate their role in fruit maintenance. Therefore, it is proposed that fruit maintenance involves kinase activity, cellular reorganization, signaling related to fruit texture formation and macromolecule synthesis.

## Conclusion

This study highlights the role of oxalate downregulation on the reprogramming of fruit physiology at translational level during post-harvest storage. The comparative analysis of proteome from wild-type and E8.2-OXDC fruits both confirmed our earlier hypothesis and more importantly identified shared and discrete pathways and distinct associations related to storage. The results obtained from the present investigation for the first time explained how low oxalate leads to the modulation of fruit quality typically linked to post-harvest storage and may reduce fruit loss due to patho-stress. Novel findings include involvement of T-complex protein eta subunit and GTP-binding nuclear protein Ran-A1 as modulator of fruit quality maintenance. Taken together, our data provide a useful basis for future work towards functional characterization of novel low oxalate responsive proteins in quality improvement program.

## Author contributions

SC: conceived the study. SC, KN, SG: designed the study. SG, KN, PA, and AS: performed the wet-lab experiments and performed proteomics analysis. SG, KN, NC, and SC: contributed to data analysis and interpretation. SC, KN, SG: wrote the manuscript.

### Conflict of interest statement

The authors declare that the research was conducted in the absence of any commercial or financial relationships that could be construed as a potential conflict of interest.
