# Supplementary material for: Comparative Proteomics of Oxalate Downregulated Tomatoes Points toward Cross Talk of Signal Components and Metabolic Consequences during Post-harvest Storage
Source: Front Plant Sci. 2016 Aug 9;7:1147. doi: 10.3389/fpls.2016.01147 (PMC4977721; doi:10.3389/fpls.2016.01147)
Supplement: Supplementary file 1 [file Table1.PDF]

Supplementary Table S1. List of unique proteins identified from wild-type fruits by MS/MS analysis

Functional Categories	Spot ID[a]	SOL ID[b]	Protein Name	Stage kinetics[c]<br>C 24h 48h 72h 96h 120h	Score	NP[d]	% coverage	Thr. Mw/ p*I*	Exp. Mw/ p*I*
**Metabolism**	*SRP 219	Solyc02g062500.2.1	2-oxoglutarate-dependent dioxygenase	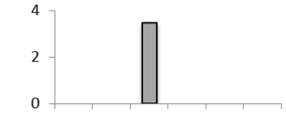	138	3	9	36.73/ 5.17	47.26/5.01
	SRP 556	Solyc01g109300.2.1	4-hydroxy-3-methylbut-2-enyl diphosphate reductase	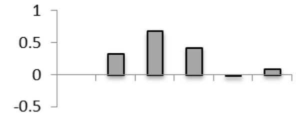	265	28	24	52.14/5.38	68.5197/ 5.62
	SRP 290	Solyc06g053200.2.1	6-phosphogluconolactonase	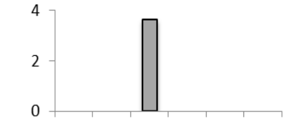	284	18	30	28.70/5.22	41.95/5.15
	*SRP 215	Solyc01g108560.2.1	Acetyl esterase	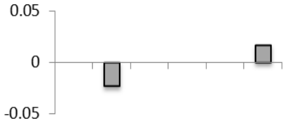	59	2	8	33.700/5.55	50.95/4.97
	*SRP 313	Solyc03g083910.2.1	Acid beta-fructofuranosidase	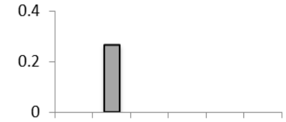	584	10	16	71.602/5.54	55.56/5.28
	SRP 507	Solyc09g092380.2.1	Adenosylhomocysteinase	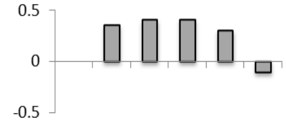	940	51	26	53.63/5.57	47.12/5.76
	*SRP 324	Solyc03g113800.2.1	Betaine aldehyde dehydrogenase	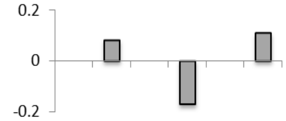	216	13	6	53.63/5.57	62.67/5.16
	SRP 169	Solyc09g009020.2.1	Enolase	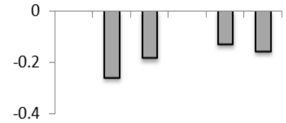	432	15	17	48.05/5.68	21.22/5.08
	SRP 220	Solyc09g064940.2.1	Phenazine biosynthesis protein PhzF family	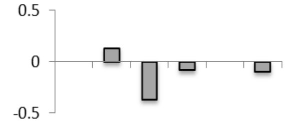	424	41	34	32.56/5.04	46.59/5.04
	*SRP 382	Solyc01g111120.2.1	Triosephosphate isomerase	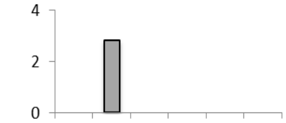	137	3	11	35.043/6.45	35.17/5.36
**Protein folding, modification, degradation**	*SRP 505	Solyc08g080790.1.1	Carboxyl-terminal proteinase	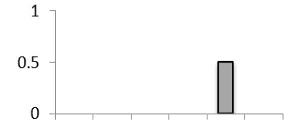	33	1	1	46.89/6.52	59.38/4.38

	SRP 212	Solyc04g015340.2.1	Serine carboxypeptidase K10B2.2	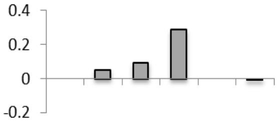	92	6	6	52.596/5.71	41.30/4.98
**Redox homeostasis**	*SRP 499	Solyc07g056490.2.1	Glutathione transferase	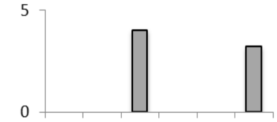	33	1	3	25.594/5.42	33.64/6.95
	*SRP 1206	Solyc01g007740.2.1	Peroxiredoxin	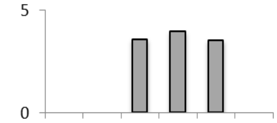	56	2	7	29.412/6.18	30.85/4.64
	*SRP 305	Solyc01g100320.2.1	Thioredoxin/protein disulfide isomerase	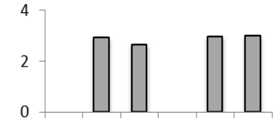	81	4	13	39.886/5.43	51.51/5.33
**Signalling**	*SRP 464	Solyc05g053620.2.1	F-box protein PP2-B1	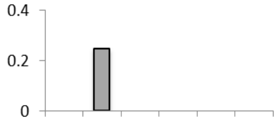	41	2	14	19.097/5.63	22.55/5.70
	*SRP 0105	Solyc01g099770.2.1	Translationally-controlled tumor protein homolog	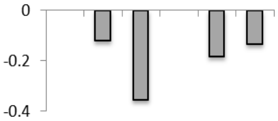	56	2	11	19.015/4.55	32.36/4.69
**Stress Response**	*SRP 878	Solyc03g093360.2.1	Universal stress protein family protein	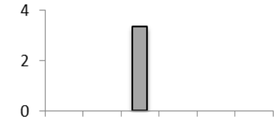	89	2	12	19.84/6.31	28.07/6.88
	SRP 1002	Solyc03g093360.2.1	Wound/stress protein	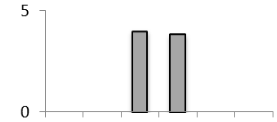	79	2	6	19.905/6.29	12.06/6.93
**Transcriptional regulator & Chromatin remodelling**	*SRP 742	Solyc02g070500.1.1	Susceptibility homeodomain transcription factor (Fragment)	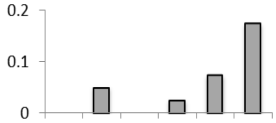	57	1	11	17.00/6.20	23.43/6.41
**Transport**	SRP 402	Solyc01g111760.2.1	V-type ATP synthase beta chain	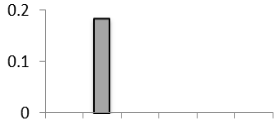	101	10	21	53.40/5.03	48.71/5.46

[a] Spot number as given on the 2-D gel images. SRP, storage responsive protein in wild-type fruits. [b] SOL Genomics Network ID. [c] Normalized protein expression value for different timepoints. [d] NP represents the number of peptides
