# Supplementary material for: Comparative Proteomics of Oxalate Downregulated Tomatoes Points toward Cross Talk of Signal Components and Metabolic Consequences during Post-harvest Storage
Source: Front Plant Sci. 2016 Aug 9;7:1147. doi: 10.3389/fpls.2016.01147 (PMC4977721; doi:10.3389/fpls.2016.01147)
Supplement: Supplementary file 2 [file Table2.PDF]

Supplementary Table S2. List of unique proteins identified from E8.2-OXDC fruits by MS/MS analysis

Functional Categories	Spot ID[a]	SOL ID[b]	Protein Name	Stage kinetics[c]<br>C 24h 48h 72h 96h 120h	Score	NP[d]	% coverage	Thr. Mw/ p*I*	Exp. Mw/p*I*
**Metabolism**	ORSRP 5	Solyc09g08 9580.2.1	1-aminocyclopropane-1-carboxylate oxidase-like protein	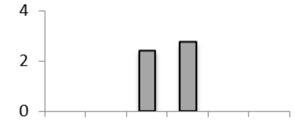	70	3	2	41.50/5. 61	22.05/ 4.57
	*ORSRP 330	Solyc07g04 4840.2.1	2 3-bisphosphoglycerate-independent phosphoglycerate mutase	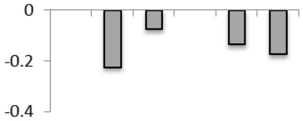	93	7	9	61.27/5. 59	65.32/5. 27
	*ORSRP 324	Solyc08g01 4130.2.1	2-isopropylmalate synthase 1	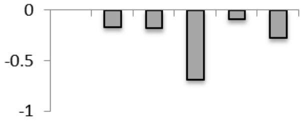	122	5	9	67.526/ 5.81	63.76/5. 16
	*ORSRP 1154	Solyc10g08 1510.1.1	5-methyltetrahydropteroyltriglutamate--homocysteine methyltransferase	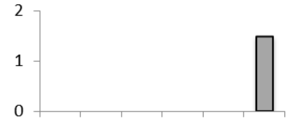	216	8	11	85.01/6. 01	80.76/5. 87
	*ORSRP 1086	Solyc09g00 9260.2.1	Fructose-bisphosphate aldolase	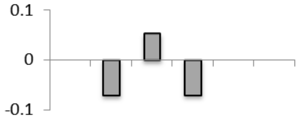	100	5	15	39.10/7. 51	53.53/6. 88
	*ORSRP 927	Solyc03g09 8240.2.1	Glutamate decarboxylase	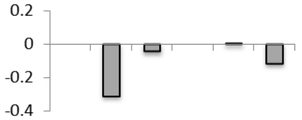	46	4	10	57.13/5. 88	63.26/6. 73
	*ORSRP 1095	Solyc01g00 5560.2.1	Isocitrate dehydrogenase	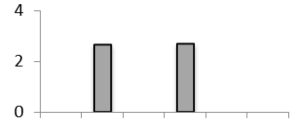	104	5	13	47.001/ 6.35	59.68/6. 25
	*ORSRP 346	Solyc08g01 3860.2.1	NAD-dependent malic enzyme 2	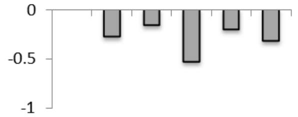	86	4	7	70.19/5. 82	65.05/5. 37
	*ORSRP 1070	Solyc01g11 0450.2.1	NADP dependent sorbitol 6-phosphate dehydrogenase	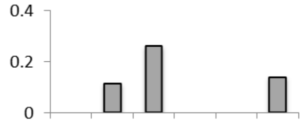	38	1	4	35.01/6. 15	49.30/5. 71
	*ORSRP 1014	Solyc10g08 0210.1.1	Polygalacturonase A	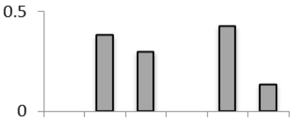	44	3	5	50.87/6. 40	60.21/5. 43

	*ORSRP 313	Solyc04g040180.2.1	S-adenosylmethionine-dependent methyltransferase	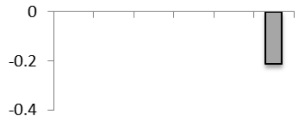	57	2	7	30.13/5.52	55.47/5.19
	*ORSRP 886	Solyc01g073640.2.1	Uncharacterized oxidoreductase Mb1385	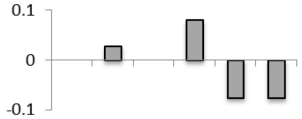	58	2	11	27.21/5.97	30.07/6.89
**Protein folding, modification, degradation**	ORSRP 23	Solyc01g101240.2.1	Aspartic proteinase	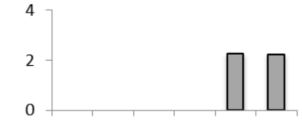	155	7	7	55.97/5.75	17.26/4.30
	*ORSRP 132	Solyc11g020040.1.1	Chaperone DnaK	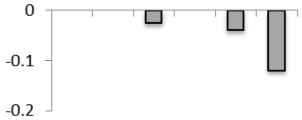	184	9	12	74.61/5.36	40.19/4.86
	ORSRP 12	Solyc06g076520.1.1	Class I heat shock protein	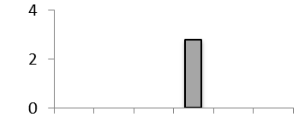	491	41	53	17.72/5.84	14.56/5.47
	*ORSRP 915	Solyc01g102960.2.1	Class IV heat shock protein	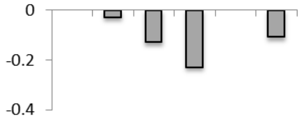	215	4	23	21.63/7.90	55.23/6.67
	ORSRP 20	Solyc12g008640.1.1	Gamma-glutamyltransferase-like protein	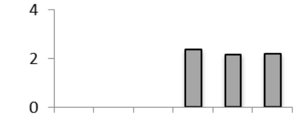	130	2	2	68.58/6.21	18.16/4.48
	*ORSRP 311	Solyc12g010040.1.1	Leucyl aminopeptidase	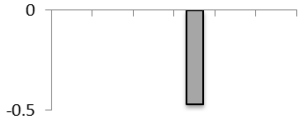	147	6	14	60.81/7.92	59.47/5.27
**Redox Homeostasis**	*ORSRP 999	Solyc05g054760.2.1	Dehydroascorbate reductase	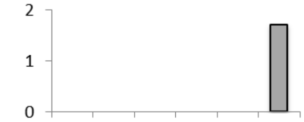	77	1	8	23.71/6.32	29.59/6.72
	*ORSRP 373	Solyc09g009390.2.1	Monodehydroascorbate reductase	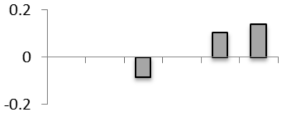	41	1	3	47.10/5.77	28.83/5.56
**Signalling**	ORSRP 70	Solyc06g052030.2.1	Importin subunit beta	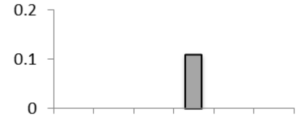	402	20	11	87.63/4.51	79.48/4.49
	*ORSRP 973	Solyc01g089970.2.1	Nucleoside diphosphate kinase	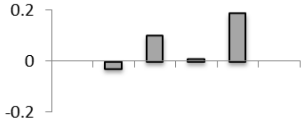	134	6	33	16.24/6.32	15.24/5.99

**Stress response**	ORSRP 11	Solyc09g090980.2.1	Major allergen Mal d 1	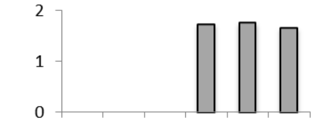	391	24	63	17.47/5.44	16.16/4.45
	ORSRP 10	Solyc04g008280.2.1	Regulator of ribonuclease activity A	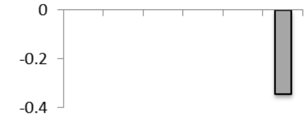	244	15	22	28.10/9.23	28.10/9.23
	*ORSRP 965	Solyc08g079170.2.1	Stress-induced protein sti1-like protein	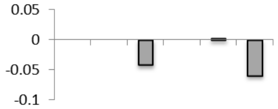	111	1	2	65.41/5.99	77.79/6.92
**Transcriptional regulator and chromatin remodelling**	*ORSRP 944	Solyc05g056250.2.1	Asparaginyl-tRNA synthetase	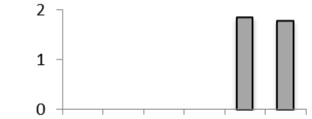	124	10	17	61.48/6.11	72.73/6.95
	*ORSRP 1159	Solyc06g065520.2.1	T-complex protein eta subunit	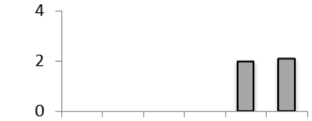	43	2	3	60.53/6.45	71.460/6.62
**Transport**	ORSRP 21	Solyc04g074230.2.1	14-3-3 protein sigma gamma zeta beta/alpha	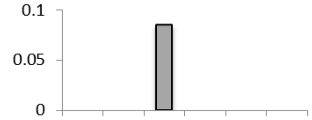	38	2	6	30.77/5.20	17.27/4.30

[a] Spot number as given on the 2-D gel images. ORSRP, oxalate decarboxylase regulated storage responsive proteins. [b] SOL Genomics Network ID. [c] Normalized protein expression value for different timepoints. [d] NP represents the number of peptides
