# Supplementary material for: Comparative Proteomics of Oxalate Downregulated Tomatoes Points toward Cross Talk of Signal Components and Metabolic Consequences during Post-harvest Storage
Source: Front Plant Sci. 2016 Aug 9;7:1147. doi: 10.3389/fpls.2016.01147 (PMC4977721; doi:10.3389/fpls.2016.01147)
Supplement: Supplementary file 3 [file Table3.PDF]

Supplementary Table S3. List of proteins common between wild-type and E8.2-OXDC fruits

Functional Categories	Spot ID[a]	Protein Name	Stage kinetics[b]
			C 24h 48h 72h 96h 120h
**Metabolism**	*SRP 904, *ORSRP 1090	Alcohol dehydrogenase 2	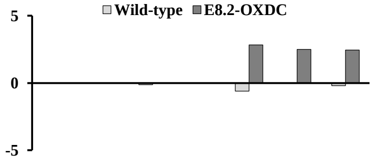
	*SRP 1070, *ORSRP 13	Glyceraldehyde-3-phosphate dehydrogenase	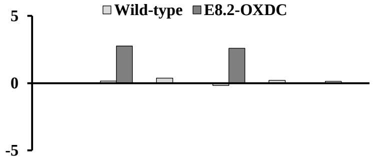
	*SRP 791, *ORSRP 329	Malate dehydrogenase	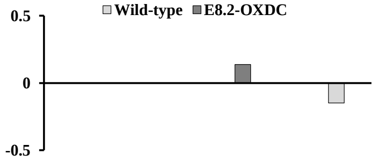
**Protein folding, modification, degradation**	SRP 822, *ORSRP 928	26S protease regulatory subunit 7	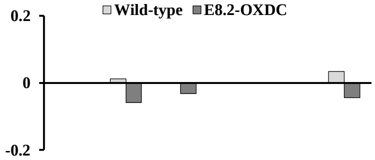
	SRP 685, *ORSRP 331	Chaperonin	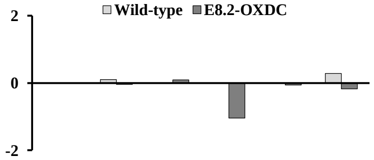
	*SRP 247, *ORSRP 296	Heat shock protein	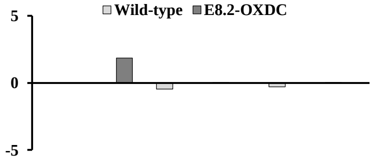
	*SRP 761, *ORSRP 876	Peptide methionine sulfoxide reductase msrA	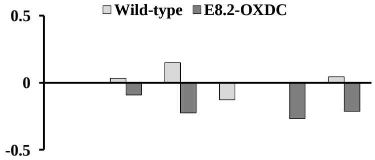
**Signalling**	*SRP 320, *ORSRP 312	Actin	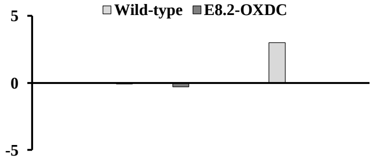
	*SRP 1030, *ORSRP 1044	GTP-binding nuclear protein Ran-A1	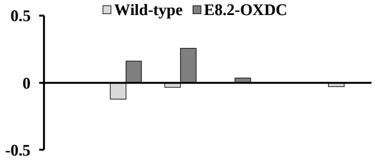

**Transcriptional regulator & Chromatin remodelling**	SRP 43, *ORSRP 959	Elongation factor beta-1	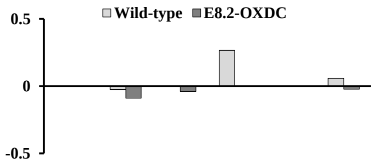
**Transport**	*SRP 308, ORSRP 57	14-3-3 protein beta/alpha-B	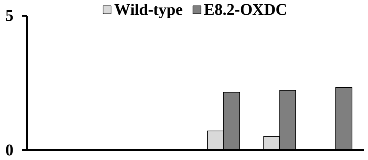

[a] Spot number as given on the 2-D gel images. [b] Normalized protein expression value from wild-type and E8.2-OXDC fruits in different timepoints.
