# Supplementary material for: Comparative Proteomics of Oxalate Downregulated Tomatoes Points toward Cross Talk of Signal Components and Metabolic Consequences during Post-harvest Storage
Source: Front Plant Sci. 2016 Aug 9;7:1147. doi: 10.3389/fpls.2016.01147 (PMC4977721; doi:10.3389/fpls.2016.01147)
Supplement: Supplementary file 5 [file Table5.PDF]

Supplementary Table S5. Genes and primers used for qRT-PCR analysis.

Spot No.	Gene description	Accession ID	Primer sequence (5´ - 3´) Forward/Reverse	Amplicon size (bp)
-	[a]18S ribosomal RNA	X51576	GGGCATTCGTATTTCATAGTCAGA/GTTCTTGATTAATGAAAACATCCT	98
SRP 507	Adenosylhomocysteinase	Solyc09g092380.2.1	TGGATCTTTGCTTTTTCCTGCTA/GGCGGCATCCATACAAGTTG	80
SRP 308	14-3-3 protein beta/alpha-B	Solyc04g076060.2.1	GGGCAGCAGTGATGAGAGAGT/CTCTCCACCAACATCCCCTATAA	70
SRP 761	Peptide methionine sulfoxide reductase msrA	Solyc03g111720.2.1	AGCAAGTCAACCACCAATCCA/AACTCCAGACCCGGCTGAT	68
ORSRP-5	1-aminocyclopropane-1-carboxylate oxidase-like protein	Solyc09g089580.2.1	ACCATGTCCTAAGCCCGATTT/TGATGCCTCCTGCGTCTGT	80
ORSRP 1014	Polygalacturonase A	Solyc10g080210.1.1	CAAGTGAAAAATGTGGTGTATGAGAAT/GGAAAGTTTGTGCTGCAATCAA	86
ORSRP 1044	GTP-binding nuclear protein Ran-A1	Solyc01g104680.2.1	AGGTGCATCCACTAGACTTCTTCA/AAACTTCTCTTGTCCGGCTGTATC	80
ORSRP 1090	Alcohol dehydrogenase 2	Solyc06g059740.2.1	GTGTCCTTAGTTGTGGAATTTCG/TGAGCCTTTTGTTGGTTTAGCA	71
ORSRP 1154	5-methyltetrahydropteroyltriglutamate--homocysteine methyltransferase	Solyc10g081510.1.1	TACATCCCCAGCAACACATTCT/TACCTAGATGGGACAGCACCAA	80

[a]Reference gene
