# Supplementary material for: Comparative Proteomics of Oxalate Downregulated Tomatoes Points toward Cross Talk of Signal Components and Metabolic Consequences during Post-harvest Storage
Source: Front Plant Sci. 2016 Aug 9;7:1147. doi: 10.3389/fpls.2016.01147 (PMC4977721; doi:10.3389/fpls.2016.01147)
Supplement: Supplementary file 6 [file Presentation1.PDF]

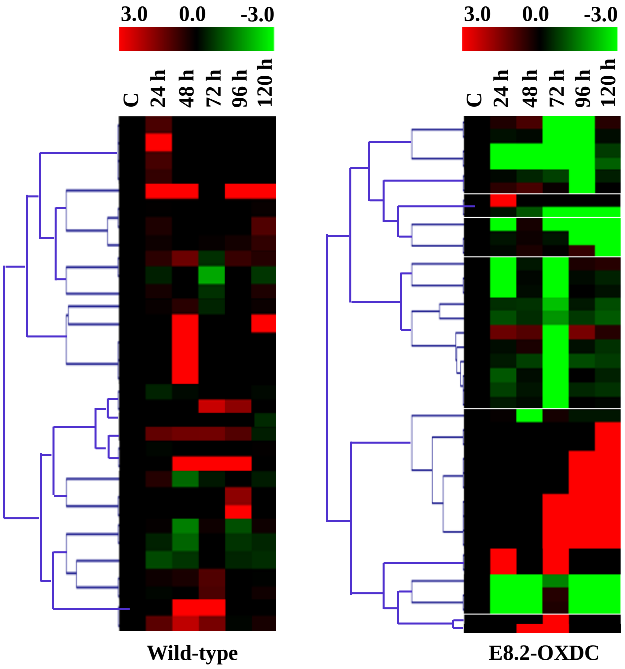

Supplementary Figure S1. Clusterogram of identified proteins. 32 and 39 proteins identified from wild-type and E8.2-OXDC were grouped into 11 clusters based on their expression pattern. The SOTA cluster tree is shown at the top, and the expression pattern in clusters are shown below. High (or low) abundance ranges from pale to saturated red (or green). C, experimental control.
