# Supplementary material for: Comparative Proteomics of Oxalate Downregulated Tomatoes Points toward Cross Talk of Signal Components and Metabolic Consequences during Post-harvest Storage
Source: Front Plant Sci. 2016 Aug 9;7:1147. doi: 10.3389/fpls.2016.01147 (PMC4977721; doi:10.3389/fpls.2016.01147)
Supplement: Supplementary file 7 [file Presentation2.PDF]

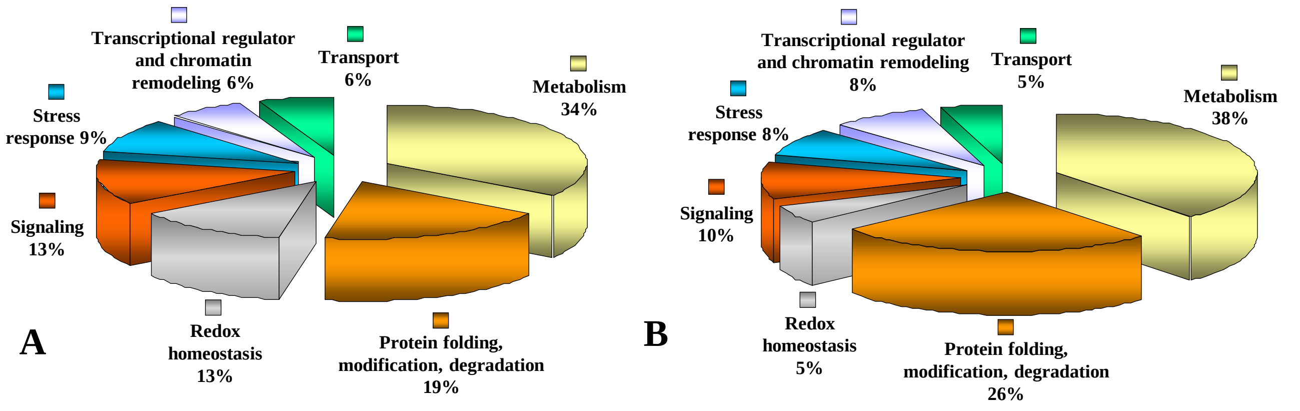

Supplementary Figure S2. Functional assignment of identified protein from wild-type and E8.2-OXDC tomato fruits. The color represents functional categories of identified proteins. Proteins were assigned a putative function using Pfam and InterPro databases and functionally categorized as represented in the pie-chart.
