# Supplementary material for: Comparative Proteomics of Oxalate Downregulated Tomatoes Points toward Cross Talk of Signal Components and Metabolic Consequences during Post-harvest Storage
Source: Front Plant Sci. 2016 Aug 9;7:1147. doi: 10.3389/fpls.2016.01147 (PMC4977721; doi:10.3389/fpls.2016.01147)
Supplement: Supplementary file 8 [file Presentation3.PDF]

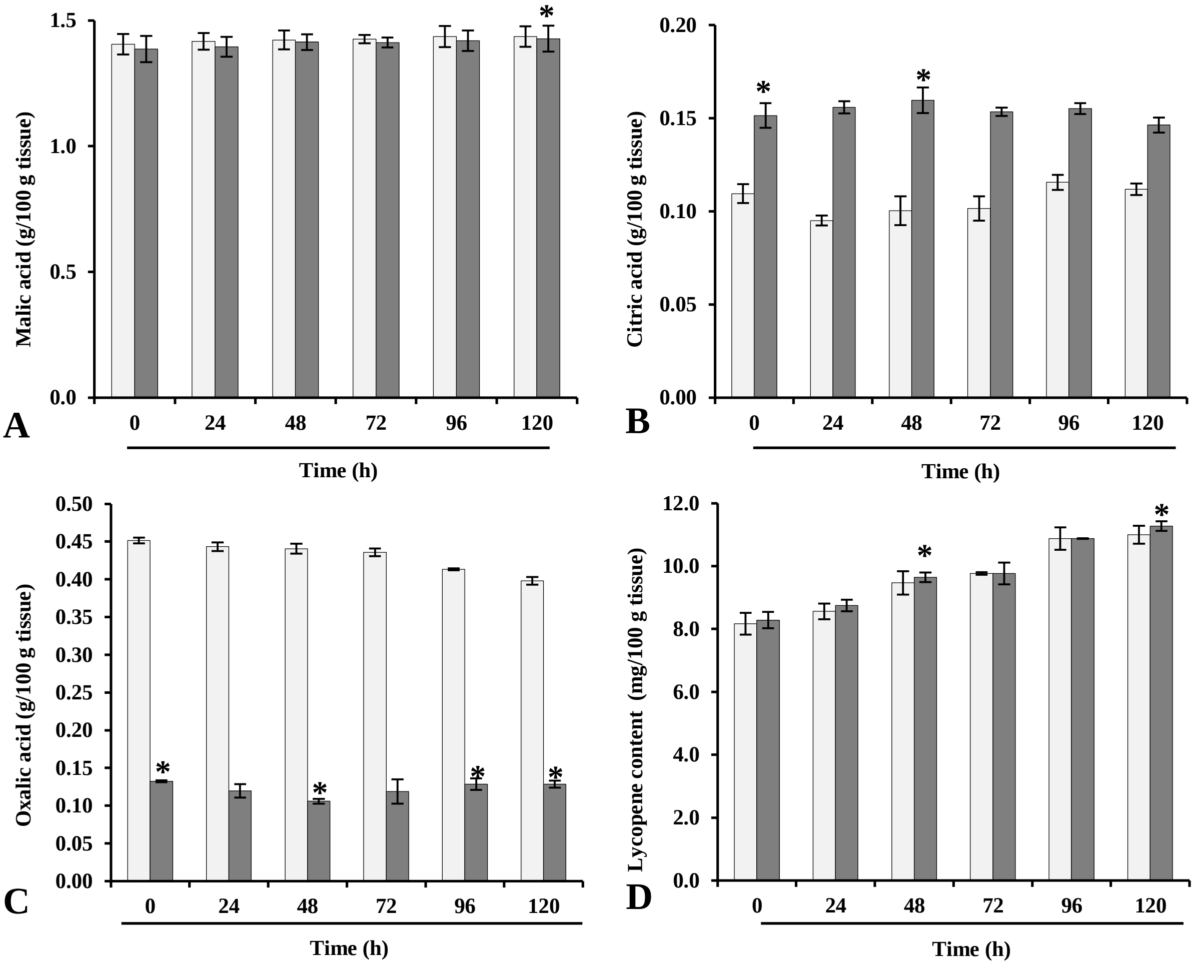

Supplementary Figure S3. Fruit quality assessment of wild-type and E8.2-OXDC tomato fruits. Estimation of (A) Malic acid, (B) Citric acid, (C) Oxalic acid, and (D) Lycopene content. Values shown are the mean ± SD from 8 fruits, Data are means of three replicates. White bar represent wild-type fruit and black bar represent E8.2-OXDC fruit. Solid line represent wild-type and dashed line represent E8.2-OXDC. Asterisk indicates significant differences among time points according to Duncan's multiple range tests ($p < 0.05$).
